# Leak-out spectroscopy as alternative method to rare-gas tagging for the Renner–Teller perturbed HCCH^+^ and DCCD^+^ ions†

**DOI:** 10.1039/d3cp04989b

**Published:** 2023-12-19

**Authors:** Kim Steenbakkers, Tom van Boxtel, Gerrit C. Groenenboom, Oskar Asvany, Britta Redlich, Stephan Schlemmer, Sandra Brünken

**Affiliations:** a Radboud University, FELIX Laboratory, Institute for Molecules and Materials Nijmegen The Netherlands sandra.bruenken@ru.nl; b Radboud University, Institute for Molecules and Materials Nijmegen The Netherlands; c I. Physikalisches Institut, Universität zu Köln Köln Germany

## Abstract

Infrared messenger-tagging predissociation action spectroscopy (IRPD) is a well-established technique to record vibrational spectra of reactive molecular ions. One of its major drawbacks is that the spectrum of the messenger-ion complex is taken instead of that of the bare ion. In particular for small open-shell species, such as the Renner–Teller (RT) affected HCCH^+^ and DCCD^+^, the attachment of the tag may have a significant impact on the spectral features. Here we present the application of the novel leak-out spectroscopy (LOS) as a tag-free method to record the *cis*-bending of the HCCH^+^ (∼700 cm^−1^) and DCCD^+^ cations (∼520 cm^−1^), using a cryogenic ion trap end user station at the FELIX laboratory. We demonstrate that the obtained LOS spectrum is equivalent to a previously recorded laser-induced reactions (LIR) spectrum of HCCH^+^. The bending modes are the energetically lowest-lying vibrational modes targeted with LOS so far, showing its potential as a universal broadband spectroscopic technique. Furthermore, we have investigated the effect of the rare gas attachment by recording the vibrational spectra of Ne- and Ar-tagged HCCH^+^. We found that the Ne-attachment led to a shift in band positions and change in relative intensities, while the Ar-attachment even led to a complete quenching of the RT splitting, showing the importance of using a tag-free method for RT affected systems. The results are interpreted with the help of high-level *ab initio* calculations in combination with an effective Hamiltonian approach.

## Abstract 2

Vibronic coupling effects in the low-lying bending modes of the open-shell linear ions HCCH^+^ and DCCD^+^ have been investigated using cryogenic infrared action spectroscopy in combination with a free electron laser.

## Graphical abstract

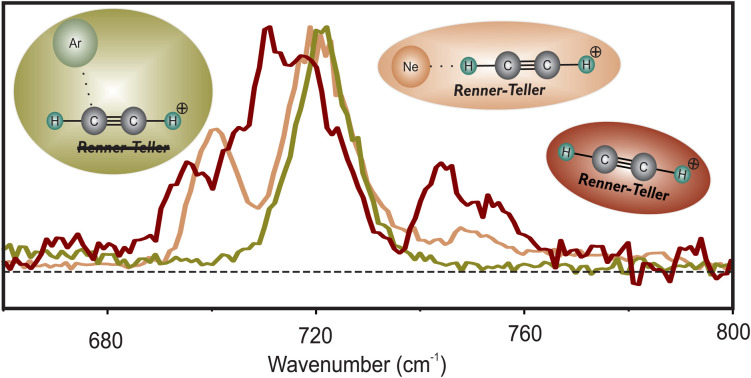

## 1 Introduction

The small hydrocarbon acetylene, HCCH, plays an important role in combustion processes^1^ and in the chemistry of exoplanetary atmospheres and the interstellar medium (ISM).^2–4^ It is proposed to be a key building block in the formation of larger organic molecules such as polycyclic aromatic hydrocarbons, *e.g.*, *via* the hydrogen abstraction-acetylene addition (HACA) mechanism in circumstellar shells,^5,6^ or *via* ion–molecule reactions in the cold interstellar medium.^7^ Acetylene itself has been detected in space,^8,9^ but its cationic counterpart, HCCH^+^, likely formed upon UV or cosmic-ray ionization in the ISM and expected to play an important part in the formation of larger hydrocarbon cations,^10–12^ has so far remained undetected.

This interesting cation, exhibiting a ^2^Π_3/2_ electronic ground state, is also relevant from a fundamental spectroscopic point of view due to its open-shell linear nature. It is subjected to both spin–orbit and Renner–Teller (RT) coupling effects complicating the rovibrational spectra of its *trans*- (ν_4_) and *cis*-bending (ν_5_) modes of which only the latter is IR active. The HCCH^+^ ion was one of the first RT affected tetra-atomic species that has been experimentally^13^ and theoretically investigated and has since been widely used as a model system.^14–19^ The spectroscopic studies on the RT affected bending modes of this ion include low- and high-resolution pulsed-field induced zero electron kinetic energy photoelectron spectroscopy (PFI-ZEKE PES),^20–22^ laser induced reactions (LIR),^23,24^ and Ar-tagging infrared predissociation (IRPD) spectroscopy.^25^ Investigations into its doubly deuterated form, DCCD^+^, are limited to two theoretical studies by Perić and Radić-Perić^26^ and Jutier and Léonard^16^ and a low-resolution photoelectron spectroscopic study by Reutt *et al.*,^27^ revealing one feature of the RT split *trans*-bending, but none of the *cis*-bending.

This abundance of spectroscopic and theoretical data makes HCCH^+^ an excellent system to test the potential of the newly developed action spectroscopic technique leak-out spectroscopy (LOS).^28^ With this technique, the mass-selected ions, stored in a low-temperature ion trap, are vibrationally excited after which they collide with a neutral buffer gas, in turn transferring their internal energy to kinetic energy. As a result of this extra kinetic energy, the ions “leak-out” of the trap and are then detected, giving rise to a nearly background-free vibrational spectrum. The LOS method is thus very universal, in particular compared to another tag-free method, laser-induced reactions (LIR^29,30^), which has also been applied previously to the *cis*-bending of HCCH^+^,^23,24^ but requires a suitable slightly endothermic reaction to be applicable. Leak-out spectroscopy has already been applied for the high-resolution ro-vibrational spectroscopy of C_3_H^+^,^28^ ro-vibrational and double-resonance rotational spectroscopy of c-C_3_H_2_D^+^, H_2_CCCH^+^, and HCCCO^+^,^31^ and to the RT inactive antisymmetric C–H stretch (ν_3_) of HCCH^+^.^32^ In these studies, the infrared excitation leading to collisional energy transfer was done in the 3 micron region, using narrow-bandwidth continuous-wave, or in some cases also ns-pulsed, radiation. Our work presented here on the low-energetic bending modes of HCCH^+^ (∼700 cm^−1^) and DCCD^+^ (∼500 cm^−1^) using a μs-pulsed infrared free-electron laser may therefore act as proof-of-principle for LOS as a broadband spectroscopic technique.

Another spectroscopic technique to record broadband vibrational spectra of reactive species under isolated conditions is IRPD action spectroscopy. This technique is based on attaching a messenger atom [usually a rare gas (RG) such as He, Ne or Ar] to the target ion at cryogenic temperatures, so that a stable, but weakly bound complex is formed, which will dissociate upon resonant single-photon absorption.^33–36^ The binding of a RG atom to a closed-shell species typically results in only minor shifts in the corresponding vibrational spectrum, such that the recorded spectrum can be taken as a proxy for that of the bare ion.^37–41^ However, for small open-shell species, such as HCCH^+^ and DCCD^+^, the interaction between the RG tag and the ion may have a significant impact on the bending potential energy curve and thus its spectral features, as seen previously for Ne-tagged HC_3_N^+^.^42^ So far, to our knowledge, no systematic study exists on the effect of the rare-gas attachment on Renner–Teller perturbed systems.

In the present work we have recorded the spectrum of the IR active *cis*-bending of HCCH^+^ with LOS and Ne- and Ar-tagging IRPD, adding to the existing LIR work of Schlemmer *et al.*^23^ and Asvany *et al.*^24^ Not only do these measurements act as proof-of-principle for the application of LOS on low-energetic vibrational modes, but they also allow for a systematic investigation of the RG attachment to RT perturbed systems. Furthermore, we have recorded the first vibrational spectrum of the DCCD^+^ ion by means of LOS and Ne-tagging IRPD. Using an effective Hamiltonian approach the observed structures were analyzed and the resulting spectroscopic constants were compared to those obtained in the earlier PFI-ZEKE PES and LIR studies.

## 2 Methods

### 2.1 Experimental methods

The experiments were performed in the cryogenic 22-pole ion trap instrument FELion located at the Free Electron Lasers for Infrared eXperiments (FELIX^43^) laboratory. A detailed account of the instrument and the employed action spectroscopic technique IRPD has been given previously.^44^ For the LOS experiments we follow the method described by Schmid *et al.*^28^ Here we give a brief account on the details specifically related to the IRPD and LOS measurements of the HCCH^+^ and DCCD^+^ ions.

**Fig. 1:**
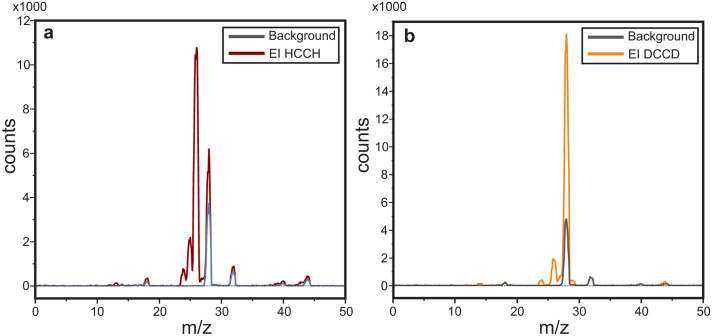
Mass spectra of ions produced from electron impact ionization (∼30 eV) of (a) HCCH and (b) DCCD overlayed with residual gas mass spectra. Here the first quadrupole functions as an ion guide from the source to the trap and the second quadrupole is used for scanning over the *m*/*z* range.

The HCCH^+^ ions are produced by electron impact (EI) ionization of acetylene (C_2_H_2_ ≥ 98%, Linde Gas) and the DCCD^+^ ions from acetylene-d2 (C_2_D_2_, 99%-d2, CDN Isotopes). Both gases were diluted with He in a 1 : 3 (precursor : He) mixing ratio to aid ionization and the pressure in the source was kept at ∼10^−5^ mbar. Fig. 1 shows the EI mass spectra (30 eV electron energy) of the HCCH and DCCD precursors overlayed with the background signal from residual gas. We dominantly form the radical cations with *m*/*z* 26 (HCCH^+^) and *m*/*z* 28 (DCCD^+^), respectively, where for the latter a small admixture of CO^+^ and N_2_^+^ is present in the same mass channel.

The ions are extracted from the source in a ∼15–50 ms long pulse and are introduced into a quadrupole mass filter to mass select the ions of interest. After mass selection the ions are guided into the 22-pole ion trap,^45^ where either an IRPD or LOS spectroscopic scheme is applied.

**Fig. 2:**
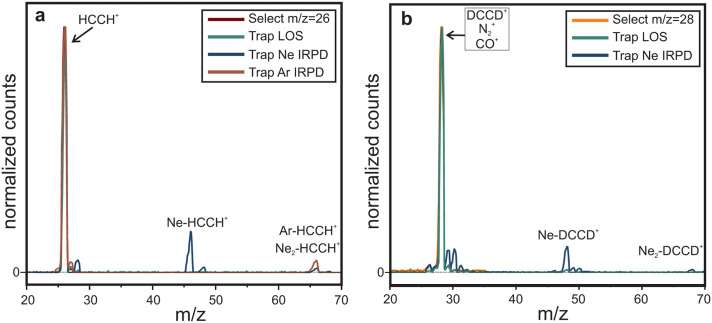
Mass selected HCCH^+^ (a) and DCCD^+^ (b) together with the mass spectra of the trap content after 600 ms trapping under Ne-tagging, Ar-tagging and leak-out conditions. Mass channels *m*/*z* +2 (*i.e. m*/*z* 28 for HCCH^+^ and *m*/*z* 30 for DCCD^+^) are a consequence of H_2_ trace contamination in the Ne. Here the first quadrupole is used to mass select the ion of interest before entering the trap and the second quadrupole is used for scanning over the *m*/*z* range after trapping.

For IRPD action spectroscopic experiments complexes of the target ions with Ne or Ar are formed inside the ion trap *via* three-body collisions by storing them at 6–7 K for Ne and 13–18 K for Ar and exposing them to a short (few 10 ms) He : Ne(Ar) pulse with high number density (∼10^15^ cm^−3^) with mixing ratio 3 : 1 for He : Ne and 5 : 1 for He : Ar. Under these conditions ∼17% of *m*/*z* 26 (*i.e.* HCCH^+^) forms a weakly bound complex with Ne and ∼5% with Ar (see Fig. 2). For *m*/*z* 28 only ∼10% binds with Ne, which is likely due to the presence of both N_2_^+^ and CO^+^ in the *m*/*z* 28 mass channel. These ions may have lower Ne binding energies than DCCD^+^, resulting in an overall lower tagging efficiency. In the trap the formed complexes are then exposed to FELIX infrared light for 1-3 s, inducing predissociation upon on-resonant irradiation. Then, the content of the trap is analyzed with a second quadrupole mass selector and a single-ion counting detector, monitoring the depletion of the ion-complex signal as a function of the FELIX wavelength.

For leak-out spectroscopy the target ions are stored and collisionally cooled in the ion trap at 15–17 K by exposing them to a short (few 10 ms) He pulse at high number density (∼10^15^ cm^−3^). The trap exit electrode potential is set so that the ions are just stored and that only a small kinetic energy gain is needed for them to leak out. These settings are continuously adjusted to account for changes in the effective voltages due to condensation. While the ions are stored, neon gas is continuously flowing into the ion trap (∼10^12^ cm^−3^) to act as a collision partner, which is needed to convert the internal energy of the excited ions to kinetic energy. The number density and trap temperature were optimized for maximum LOS signal strength and avoidance of formation of Ne-complexes. In the trap, the bare ions are exposed to FELIX infrared radiation for 1–3 s and leak-out upon on-resonant irradiation. After each cycle the trap content is discarded and the process repeats. To record the vibrational spectrum one may either monitor the depletion of the bare-ion signal as a function of the FELIX wavelength after each trapping cycle, or directly monitor the ions leaking out during the trapping period. In this work the former method was used.

The 440–600 cm^−1^ and 650–840 cm^−1^ wavenumber ranges were covered in this study using the free-electron IR laser FEL-2 of the FELIX Laboratory‡‡https://www.ru.nl/en/hfml-felix. with a macropulse repetition rate of 10 Hz, maximum pulse energy in the trap region of <20 mJ (at 700 cm^−1^), and a full width at half maximum (FWHM) of around 0.7% of the center wavenumber. To account for varying laser pulse energy *E*, pulse number *n*, and for saturation effects, the signal is normalized prior to averaging using 
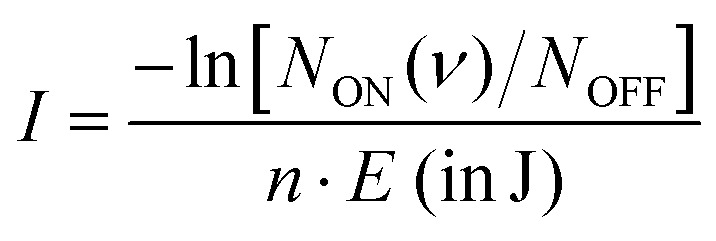
, giving the intensity *I* in units of cross-section per joule as a function of the frequency (*ν*), where *N*_ON_ (*ν*) gives the on- and *N*_OFF_ the off-resonance ion count. The individual measurements are averaged using statistical binning with a bin size of 1 cm^−1^ to obtain the final spectrum. The final spectra were normalized with maximum intensity equal to one, to facilitate comparison between different methods.

### 2.2 Theoretical methods

#### 2.2.1 *Ab initio* calculations

*Ab initio* quantum chemical calculations were used to optimize the geometric structure and calculate the harmonic vibrational frequencies of the bare HCCH^+^ and DCCD^+^ species and to compute the potential energy curve of the Ne- and Ar-tag around the HCCH^+^ ion. All calculations were performed at the partially spin-restricted coupled cluster level of theory, with single, double, and perturbative triple excitations [RCCSD(T)].^46^ For geometry optimization and subsequent harmonic wavenumber calculations on the HCCH^+^ and DCCD^+^ ions Dunning's aug-cc-pV5Z basis set^47–49^ was used. To investigate the interaction of HCCH^+^ with the Ne and Ar atom a one-dimensional cut of the potential energy curve (aug-cc-pVTZ)^47–49^ was made in Jacobi coordinates by attaching the Ne/Ar atom to the center of mass of the HCCH^+^. In these calculations the attachment angle was kept fixed while all other geometry parameters were allowed to relax. The computed interaction energies were counterpoise corrected to account for the basis set superposition error. All quantum chemical calculations were performed using the MOLPRO suite, version 2015.1.^50^

#### 2.2.2 Effective Hamiltonian

To simulate the spin-vibronic levels of HCCH^+^ and DCCD^+^ an effective Hamiltonian approach was used similar to that of Yang and Mo,^20^ Tang *et al.*^21^ and Steenbakkers *et al.*^42^ The chosen basis is given by:

1 |***n****PJM*;*p*〉

where *J* is the total angular momentum quantum number, *M* and *P* are quantum numbers corresponding to projection of the total angular momentum on the space-fixed and molecule-fixed axes, respectively, *p* = ±1 is the parity, and

2 |***n***〉 = |*Λ*〉|*SΣ*〉|*v*_5_*l*_5_〉|*K*〉.

Here, *S* = 1/2 is the total electron spin quantum number and *v*_5_ = 0, 1,… is the vibrational quantum number of the *cis*-bending mode ν_5_. We only include the *cis*-bending mode since the *trans*-bending mode (ν_4_) is IR inactive. Coupling with the symmetric (ν_1_) and anti-symmetric C–H (ν_3_) stretch as well as with the C–C stretch (ν_2_) were ignored. Furthermore, *Λ* = ±1, *Σ* = ±1/2, and *l*_5_ = −*v*_5_, −*v*_5_ + 2,…,*v*_5_ are the quantum numbers corresponding to projection on the molecular axis of the electronic orbital, total electron spin, and vibrational angular momenta, respectively. Quantum number *K* = *Λ* + *l*_5_ represents the projection of the total angular momentum excluding electron spin and *P* = *K* + *Σ*. Quantum numbers *J*, *M*, and *p* are good quantum numbers and the Hamiltonian is independent of *M*. In our model (excluding rotation) *K* and *P* are also good, since we only include diagonal spin–orbit coupling and first order RT and neglect Coriolis coupling. Because of this approximation states with parity *p* = +1 and *p* = −1 are degenerate, and we can compute the Hamiltonian by restricting the basis to *P* > 0, without parity adapting the basis. For a basis truncated at *v*_5_ = 10 we find that energy levels are converged up to *v*_5_ = 4.

We approximate the total effective Hamiltonian by:

3 *Ĥ* = *Ĥ*_vib_ + *Ĥ*_SO_ + *Ĥ*_RT_ + *Ĥ*_rot_,

where *Ĥ*_vib_ represents the vibrational energy,4
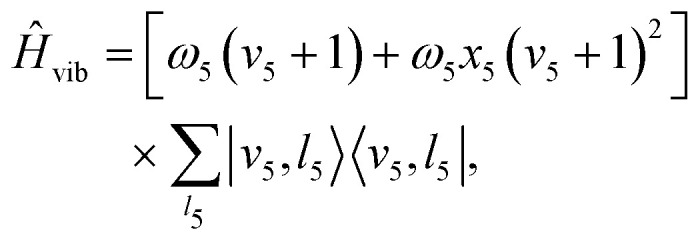
with *ω*_5_ the harmonic frequency of the *cis*-bending modes ν_5_ and *x*_5_ its anharmonic correction. The spin–orbit Hamiltonian is given by^51^

5 *Ĥ*_SO_ = *A*_SO_*L̂*_*z*_*Ŝ*_*z*_

where *A*_SO_ represents the spin orbit constant and *L̂*_*z*_ and *Ŝ*_*z*_ the molecule fixed components of the electronic orbital and spin angular momenta operators, respectively. The effective RT Hamiltonian is:^52^6
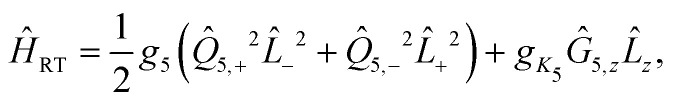
where the constant *g*_5_ is related to the dimensionless RT constants *ε*_5_ as

7 *g*_5_ = *ε*_5_*ω*_5_.

In this work *ω*_5_ and *ε*_5_ are either fitted or approximated from *ab initio* harmonic frequency calculations as8
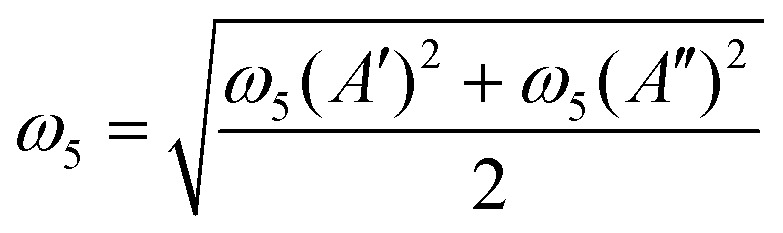
and9
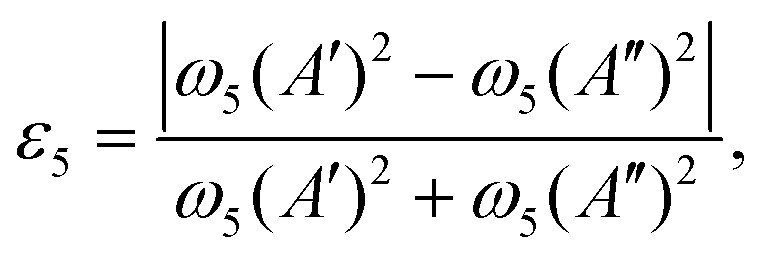
where *ω*_5_(*A*′) is the *ab initio* harmonic frequency of the in-plane and *ω*_5_(*A*′′) of the out-of-plane *cis*-bending. Here eqn (8) and (9) are derived for a first order Renner–Teller problem described in ref. 53.

The complex normal mode operators *Q̂*_5,±_ are related to the Cartesian normal modes *Q̂*_5,*x*_ and *Q̂*_5,*y*_ by

10 *Q̂*_5,±_ = *Q̂*_5,*x*_ ± *iQ̂*_5,*y*_

and *L̂*_±_^2^ represent the orbital angular momentum ladder operators coupling states *Λ* = 1 and *Λ* = −1. Finally, the *g*_*K*_5__ term, first introduced by Brown,^52^ is a correction to the RT Hamiltonian as a result of the *K*-dependent mixing of the vibrational levels of a Π electronic state with the vibrational levels of Σ and Δ states. This term is dependent on the molecule fixed components of the electronic orbital (*L̂*_*z*_) and vibrational angular (*Ĝ*_5,*z*_) momenta.

Finally, the rotational Hamiltonian is given by

11 *Ĥ*_rot_ = *BR*^2^ ≈ *B*[(*Ĵ*^2^ − *Ĵ*_*z*_^2^ + *Ŝ*^2^ − *Ŝ*_*z*_^2^ − *Ĝ*_z_^2^) − (*Ĵ*_+_*Ŝ*_−_ + *Ĵ*_−_*Ŝ*_+_)],

where *B* is the rotational constant. In this equation the final two terms take *P*-state mixing into account rendering *P* to not be a good quantum number. For the Σ states this interaction is strongest, while for the Π and Δ states it is moderate for the lower *J*-states. From this point on we will therefore label the Π and Δ states with their largest *P*-state contribution (as approximated by the expectation value of *J*_*z*_).

The wave function is expanded in the basis12
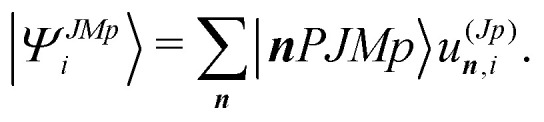
The expansion coefficients *u*^(*Jp*)^_***n***,*i*_ are determined variationally by solving the matrix eigenvalue problem given in eqn (13) below, using the free and open source numerical software Scilab version 6.1.1.^54^

13 ***Hu***_*i*_ = *E*_*i*_***u***_*i*_,

where *E*_*i*_ are the eigenvalues and ***H*** the Hamiltonian matrix of which the matrix elements are given in ref. 55. The population of the computed energy levels is then approximated with the Boltzmann distribution.

The line strengths for the Δ*K* = ±1 transitions are computed by14
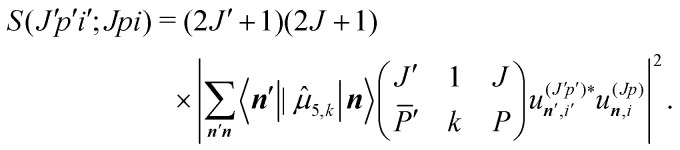
A derivation of this expression is given in the ESI,† where we also define the “time-reversal notation” for *P̄*′ in the 3−*j* symbol. This derivation of the line intensity is based on literature and text-book results. It is provided for the convenience of the reader, and to define exactly how the calculation was done. The dipole operator *μ̂*_*k*,5_ is approximated by

15 *μ̂*_5,*k*_ = *μ̂*_5,±_ = *μ*^⊥^_5_*Q̂*_5,±_.

The perpendicular dipole moment, *μ*^⊥^_5_, is set equal to 1, since all computed intensities are normalized and only one vibrational mode is considered.

## 3 Results

### 3.1 Leak-out spectroscopy (LOS) of HCCH^+^

**Fig. 3:**
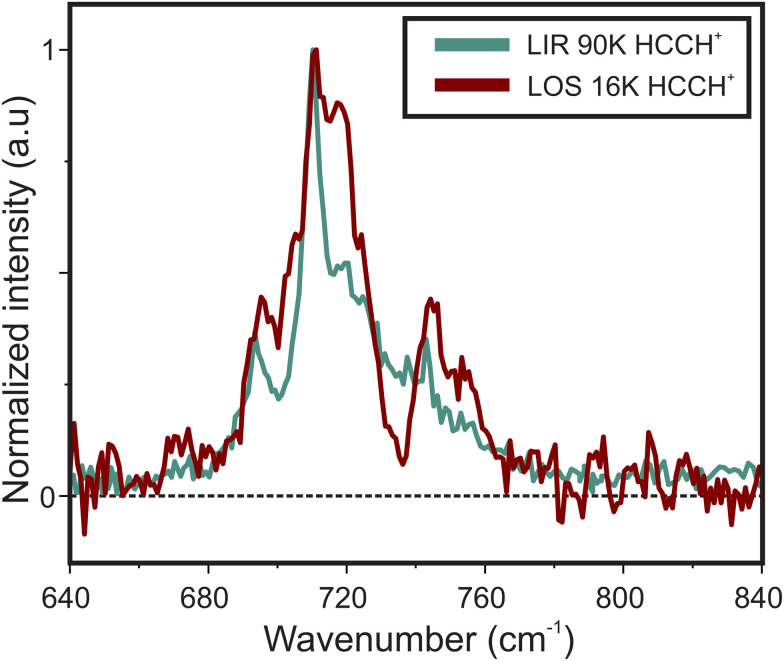
Comparison of the infrared spectra of the *cis*-bending of HCCH^+^ taken with LOS (in red) and in previous work with LIR^23^ (in green) recorded at a nominal trap temperature of 16 and 90 K, respectively.

**Fig. 4:**
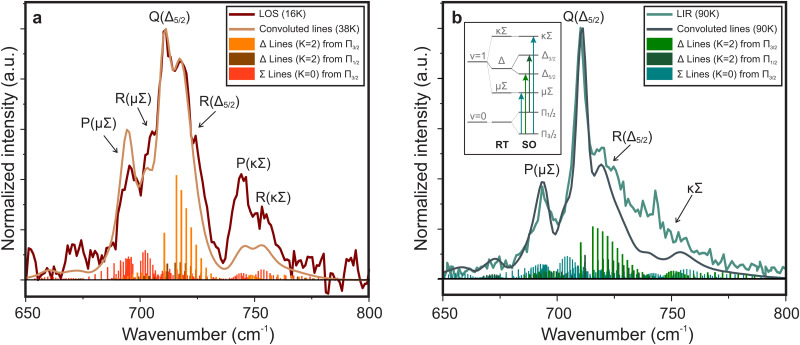
Infrared spectra simulated with the effective Hamiltonian model described in Section 2.2.2. The spectroscopic parameters were taken from Tang *et al.*^21^ and the temperature and FWHM were fitted to the spectra obtained with LOS (a) and LIR (b),^23^ recorded at a nominal trap temperature of 16 K and 90 K, respectively. The best fit internal temperature is 38 K for the LOS and 90 K for the LIR measurement. The corresponding spectroscopic and fit parameters are given in Table 1. The inlay in (b) shows an illustrated energy level diagram (not to scale).

Fig. 3 shows the spectrum of the *cis*-bending mode of the HCCH^+^ ion recorded with LOS overlayed with the previously recorded LIR spectrum.^23^ The positions of the most intense features match within the experimental uncertainty of the band center (∼2 cm^−1^, taking into account the wavenumber calibration), but a clear difference is observed in the band profiles. To explain these differences the ro-vibrational spectra were simulated with the effective Hamiltonian approach described in Section 2.2.2. The spectroscopic constants were kept fixed to experimentally obtained values from Tang *et al.*^21^ and the temperature and line width (FWHM) of individual ro-vibrational lines were adjusted (“fitted”) by hand to match best the observed band profile (see Table 1). The resulting simulations (shown in Fig. 4) are in good agreement with the experimental spectra, though the intensities of the RT affected Σ bands are either over-predicted (for the μΣ or under-predicted (for the κΣ)). This may be a consequence of (i) neglecting coupling to other vibrational modes in our model, (ii) a potential discrepancy between the collisional cross-sections of the μΣ and κΣ modes or (iii) the sensitivity of the leak-out efficiency possibly leading to effects in the intensity of the vibrational energy (see Section 3.2.1 for further discussion). High-level quantum dynamical computations are thus likely needed to interpret the observed intensities. Using the simulated spectra the observed band profiles can be explained and the most intense bands in both spectra can be assigned to belong dominantly to the μΣ, Δ_5/2_, and κΣ transitions from the Π_3/2_ vibrational ground state at approximately 700, 712, and 750 cm^−1^, respectively. Furthermore, the transition to the Δ_3/2_ state from the excited Π_1/2_ spin–orbit state is about 2 cm^−1^ shifted from the Π_3/2_–Δ_5/2_ band and will contribute to the 712 cm^−1^ feature. These transitions obey the Δ*K* ± 1 and Δ*P* = ±1 selection rules (see panel in Fig. 4), of which the latter is somewhat relaxed due to the *P*-state mixing. Clearly, the rotational structures of these bands overlap, complicating the assignment of the convoluted spectra. At higher *T*, this convolution becomes more problematic and can be attributed to several causes. First, more *J* levels are occupied resulting in an effective “smearing” of the substructure. Second, with the occupation of higher *J*-levels the *P*-state mixing becomes more prevalent, loosening the Δ*P* = ±1 selection rule. Finally, the contribution of transitions from the Π_1/2_ excited state (∼30 cm^−1^ above the ground state) starts to increase: at 38 K (simulation temperature for the LOS experiment) this contribution, determined assuming a Boltzmann distribution, is 26.5%, while at 90 K (LIR experiment) it is 40.2%. A full list of calculated transitions with their respective intensities can be found in ESI:† Table S1 for the Σ and ESI:† Table S2 for the Δ states.

**Table 1:** Spectroscopic and experimental parameters used for the simulated LOS and LIR spectra of HCCH^+^ shown in Fig. 4. Unmarked parameters are taken from ref. 21

	*ω*_5_ (cm^−1^)	*ε*_5_	*x*_5_	*A*_SO_ (cm^−1^)	*g*_*K*_5__ (cm^−1^)	*B* (cm^−1^)	*T* (K)	FWHM (cm^−1^)
HCCH^+^ LOS	719.39(35)	−0.02737(10)	−0.00373(18)	−31.283 (66)	4.545 (47)	1.1028 (11)	38^a^	5.5^a^
HCCH^+^ LIR	719.39(35)	−0.02737(10)	−0.00373(18)	−31.283 (66)	4.545 (47)	1.1028 (11)	90^a^	4.5^a^
DCCD^+^ LOS	528.5^a^	−0.02737(10)	−0.00373(18)	−31.283 (66)	2.3^a^	0.80^b^	27^a^	3^a^

a Parameters are fitted by hand.

b Parameters are calculated *ab initio* on RCCSD(T)/aug-cc-pV5Z level of theory.

The good match of the experimental infrared spectra with the simulations using the spectroscopic parameters obtained by Tang *et al.*^21^ provides some clarification regarding the discrepancies between the Renner–Teller parameters for the *cis*-binding mode reported in the three experimental works: Yang and Mo^20^ reported *ω*_5_ = 704.05 cm^−1^ and *ε*_5_ = 0.0219, Tang *et al.*^21^*ω*_5_ = 719.39 cm^−1^ and *ε*_5_ = −0.0274 and Schlemmer *et al.*^23^*ω*_5_ = 710 cm^−1^ and *ε*_5_ = 0.032. Most striking is the significant difference between spectroscopic parameters obtained by Yang and Mo^20^ and Tang *et al.*,^21^ since both works use the same, high-resolution, experimental technique (zero-kinetic-energy photo-electron spectroscopy). The effective Hamiltonians used for the fitting were also very similar with the largest difference being the inclusion of an anharmonicity term and the Brown *g*_*K*_5__ Renner–Teller correction by Tang *et al.*^21^ We have simulated the vibrational spectra using both Hamiltonians with their corresponding parameters and found a far better agreement with the parameters of Tang *et al.*,^21^ where the inclusion of the *g*_*K*_5__ term turned out to be critical to fit our experimental data.

It is noteworthy that the fitted rotational temperature of the LOS spectrum (38 K) is significantly higher than the nominal trap temperature (16 K), while for the LIR spectrum this is not the case (both recorded and fitted temperatures are 90 K). This effect of the LOS technique has been previously observed by Asvany *et al.*,^31^ where they discuss that collisions of the ions with heaver species (*i.e.* Ne) in an RF field leads to heating of the stored ions. The line width is expected to be dominated by the resolution of the free-electron laser whose FWHM may vary between 0.6–0.8% of the center wavenumber, corresponding to Δ*ω* = 4.2–5.6 cm^−1^ at 700 cm^−1^ and Δ*ω* = 3–4 cm^−1^ at 500 cm^−1^. The fitted FWHM are in agreement with these values. The fact that the structure of both the LIR and LOS spectra can be reproduced by the simulation at different temperatures, indicates that the discrepancy between the obtained spectra is mainly a result of the temperature difference. Furthermore, the simulations show that recording a vibrational spectrum at different temperatures may be of great help in assigning the observed structure and validating the used model, especially for a relatively low-resolution technique.

### 3.2 Leak-out spectroscopy of DCCD^+^

**Fig. 5:**
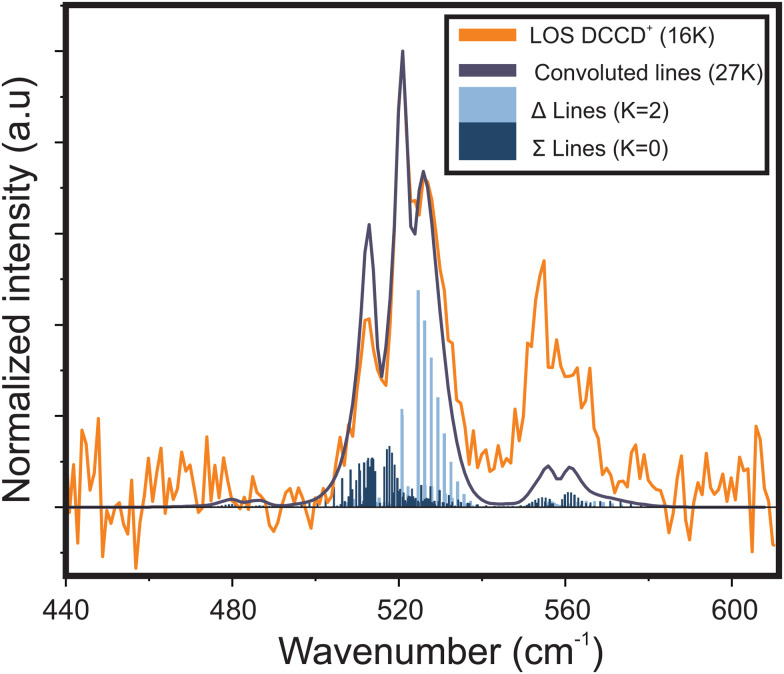
Vibrational infrared spectrum of the *cis*-bending of DCCD^+^ recorded by means of LOS at a nominal trap temperature of 16 K. The experimental spectrum is overlayed with a spectrum simulated with the effective Hamiltonian model described in Section 2.2.2 using a best fit internal temperature of 27 K. Used spectroscopic parameters are given in Table 1.

Expanding on this proof-of-principle experiment, LOS was used to record the first vibrational spectrum of the RT-perturbed *cis*-bending mode of DCCD^+^, after which it was simulated with the model described above (see Fig. 5). Regarding the spectral shape a similar band profile is observed compared to that of the HCCH^+^ ion (an overlay of the structures is given in the ESI:† Fig. S1). Since isotopic substitution should not alter the electronic structure of the ion the *A*_SO_, *ε*_5_ (=*g*_5_/*ω*_5_) and *x*_5_ parameters were kept fixed to those of HCCH^+^. The rotational constant *B* = 0.80 cm^−1^ was calculated the *ab initio* at RCCSD(T)/aug-cc-pV5Z level of theory and the parameters that are dependent on the ion mass (*ω*_5_ and *g*_*K*_5__), temperature and line width were fitted to the band contour. The best fit spectroscopic and experimental parameters are given in Table 1.

From *ab initio* calculations the harmonic vibrational frequency and Renner–Teller constant can be extracted for both species. Our calculations at RCCSD(T)/aug-cc-pV5Z level of theory give *ω*_*cis*_ = 725.2/736.2 cm^−1^ (*ω*_5_ = 730.7 cm^−1^, *ε*_5_ = 0.015) for HCCH^+^, and *ω*_*cis*_ = 533.4/540.7 cm^−1^ (*ω*_5_ = 537.0 cm^−1^, *ε*_5_ = 0.014) for DCCD^+^. For both species the calculated harmonic frequency is overestimated compared to the fitted value, but the ratio between the two matches very well: 
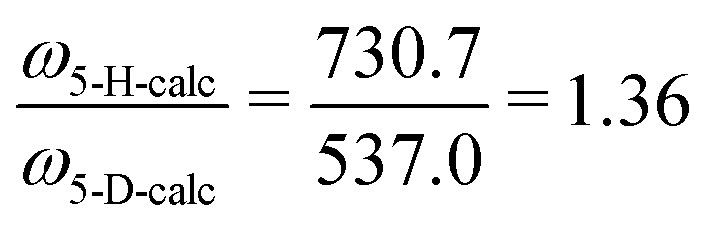
 and 
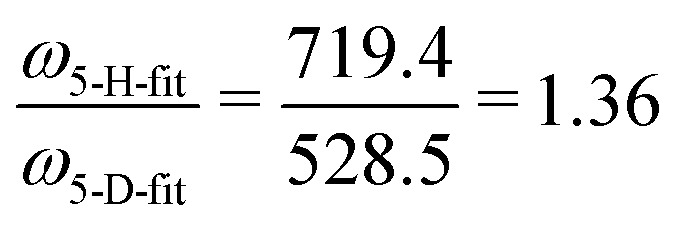
.

The *g*_*K*_5__ parameter, which captures the mixing of the excited ^2^Σ in the ground ^2^Π electronic state upon the bending of the ion, decreases with deuterium substitution. This is in line with previous findings of Smith *et al.*^56^, who investigated deuterium substitution in SiCH and found that 
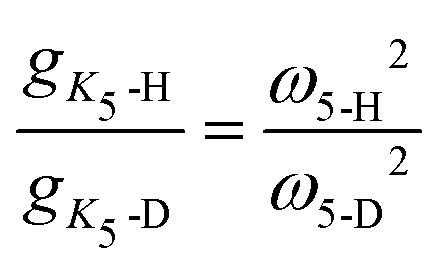
. This equation only holds when the coupling and energy gap between the excited ^2^Σ in the ground ^2^Π is isotopically invariant. From the fit we get 
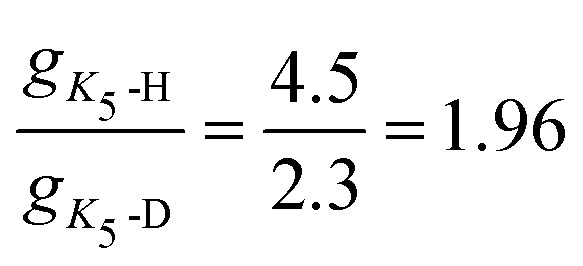
 and 
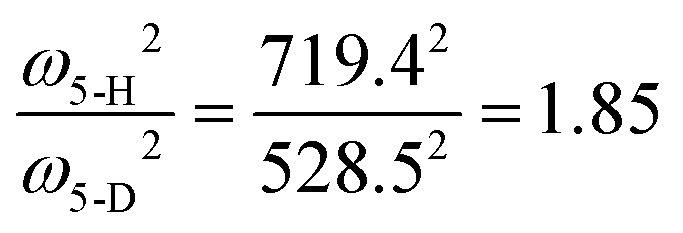
, which is in reasonable agreement considering that the DCCD^+^ parameters were fitted by hand. This agreement indicates that the isotope substitution of HCCH^+^ indeed only has a minimal impact on the electronic landscape and shows that isotope substitution can be extremely useful in understanding the complex ro-vibrational structure of Renner–Teller affected species and can aid in justifying the used model and parameter space.

Since this is the first vibrational spectrum of DCCD^+^ a comparison with earlier theoretical works is imperative. Table 2 shows the rotational band origins of the four vibrational bands of ν_5_ (μ^2^Σ_1/2_, ^2^Δ_5/2_, ^2^Δ_3/2_, and κ^2^Σ_1/2_) obtained from the fit together with the *ab initio* values of earlier theoretical work by Perić and Radić-Perić^26^ and Jutier and Léonard.^16^ Both works are based on a variational approach, where Perić and Radić-Perić used reduced degrees of freedom and Jutier and Léonard a full set of valence coordinates. Though both works report fairly different values, the divergence from the experimental work, here captured by the root-means-squared (RMS) of the difference between the calculated and observed values, is almost equal. The largest deviations are found for the RT affected ^2^Σ_1/2_ states, where Perić and Radić-Perić^26^ underestimate and Jutier and Léonard^16^ overestimate the Renner–Teller splitting. Furthermore, both works overestimate most of the predicted band positions.

**Table 2:** Comparison of rotational band origins of *cis*-bending mode ν_5_ of DCCD^+^ (in cm^−1^)

State	Perić^26^ a (o-c)^b^	Jutier^16^ (o-c)^b^	Our work
μ^2^Σ_1/2_	530(−15)	512(3)	515
^2^Δ_5/2_	532(−7)	530(−5)	525
^2^Δ_3/2_	560(−4)	559(−3)	556
κ^2^Σ_1/2_	562(−5)	574(−17)	557
RMS	9	9	

a The effect of the spin–orbit coupling (ignored in ref. 26) was added to the rovibronic energies to facilitate comparison to ref. 16, where SO was included.

b The values between brackets represent the difference between the observed and calculated band origins.

#### 3.2.1 Discussion on measured and calculated intensities

Finally, we wish to draw attention to the low energy of the recorded *cis*-bendings of HCCH^+^ and DCCD^+^. As described by Schmid *et al.*,^28^ the LOS signal should critically depend on the energy of the vibrational transition and may depend on the ion temperature as well. In principle, the kinetic energy release is divided among the two collision partners, here Ne (*m* = 20 u) and HCCH^+^ (*m* = 26 u) or DCCD^+^ (*m* = 28 u), resulting in a maximum energy gain of 
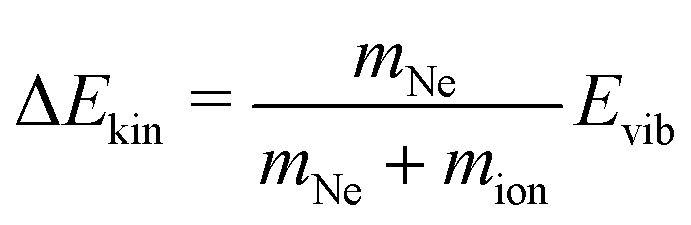
 for the ion. Therefore, with lower *E*_vib_ also Δ*E*_kin_ will decrease. The *cis*-bending of HCCH^+^ has a vibrational energy of ∼700 cm^−1^ (∼0.087 eV) and will consequentially result in a maximum kinetic energy gain of only ∼300 cm^−1^ or ∼0.037 eV. For the case of DCCD^+^ the vibrational energy is even lower (∼520 cm^−1^) resulting in a maximum gain of ∼216 cm^−1^ (∼0.027 eV). To date, these bending modes are by far the lowest-energy vibrational modes recorded with LOS and act as proof-of-principle for obtaining broadband vibrational spectra, showing the capabilities of this technique in combination with the FELIX free electron lasers.

To determine the effect of the vibrational energy on the observed leak-out intensity one needs to take the height of the trapping barrier into account. Ideally the height of the trapping barrier is set so that (almost) all ions are trapped but can still leak-out upon resonant irradiation. The setting of the trap barrier therefore affects the leak-out efficiency as shown by Bast *et al.*^57^ As a consequence, a lower gain of kinetic energy would reduce the leak-out intensity. In order to find out to what extent intensity variations within a band are influenced by this effect the intensities of the *cis*-bending vibrations for HCCH^+^ at around 700 cm^−1^ and DCCD^+^ at around 520 cm^−1^ should be carefully analyzed. Only then one could judge whether a significant intensity variation within one of the above-mentioned bands could be associated with a changing efficiency of the leak-out process. Since LOS is a considerably new method many such systematic studies are needed to get a better account on its limits.

Notably, the discrepancies between the measured and calculated intensities within the *cis*-bending vibrations of HCCH^+^ and DCCD^+^ only occur in the Sigma bands. This is an indicator that the deviation might be associated with an incomplete theoretical treatment, *e.g.*, by ignoring couplings to other modes. Also here more systematic studies are needed to explain the current findings.

### 3.3 Infrared predissociation spectroscopy of HCCH^+^

**Fig. 6:**
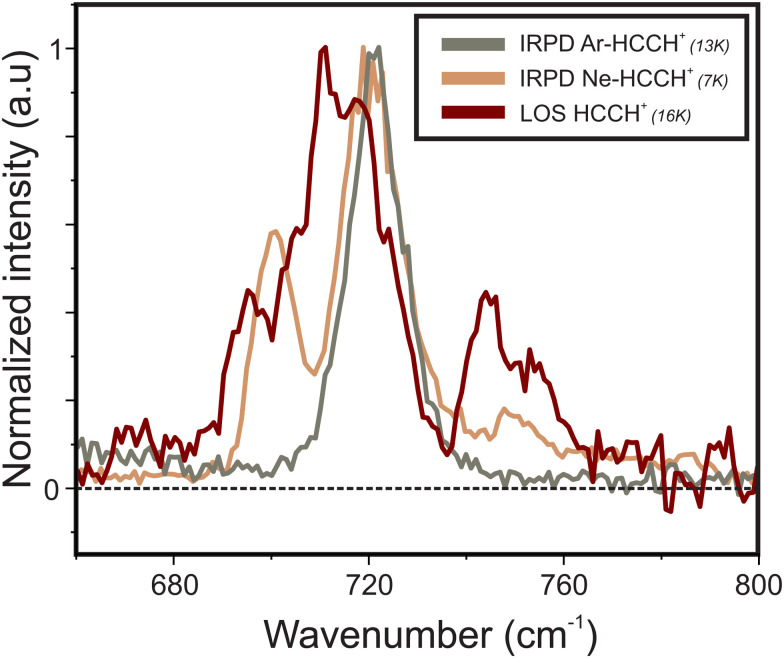
Comparison of the infrared spectra of the bare HCCH^+^ ion taken with LOS and of Ne–HCCH^+^ and Ar–HCCH^+^ taken with IRPD.

The well-established infrared predissociation action spectroscopy (IRPD) provides an alternative method to record the spectra of small reactive ions. With this technique a messenger atom is weakly bound to the ion and its vibrational spectrum is taken as a proxy for that of the bare ion. We recorded vibrational spectra of HCCH^+^ with the IRPD method using Ne and Ar as tags. Earlier work on the Ar-tagged IRPD spectrum of HCCH^+^ by Relph *et al.*^25^ did not allow for an analysis on the Renner–Teller structure due to the low signal-to-noise in the 600–1000 cm^−1^ region. The resulting spectra are shown in Fig. 6 together with the LOS spectrum of HCCH^+^, previously discussed in Section 3.1.

**Fig. 7:**
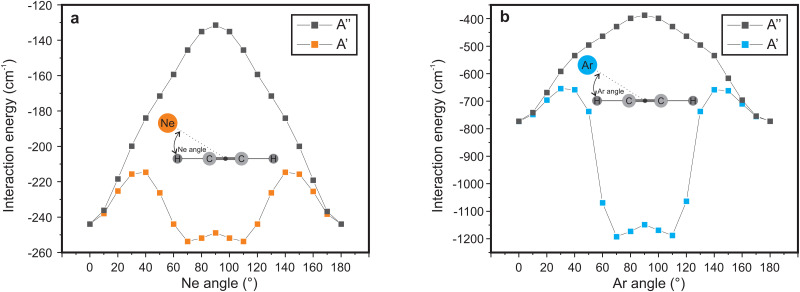
Calculated counterpoise corrected interaction energies as a function of (a) Ne angle and (b) Ar angle with respect to the molecular axis. The symmetric *A*′ state corresponds to configuration (π_*x*_)^1^(π_*y*_)^2^ and the antisymmetric *A*′′ state to (π_*x*_)^2^(π_*y*_)^1^.

Very clear differences can be observed between the three spectra: first, for both IRPD spectra no rotational substructure is observed due to the decrease of the rotational constant upon rare-gas attachment. Second, the Ne-tag induces a shift in band position together with a change in relative intensity altering the Renner–Teller pattern. The Ar-tag results in a collapse of this structure, indicating a complete quenching of the Renner–Teller splitting. To explain this behavior, we performed calculations on the geometrical structure of the two RG–HCCH^+^ complexes. A scan of the potential energy curve was computed at RCCSD(T)/aug-cc-pVTZ level of theory, where the Ar or Ne atom were displaced around the center of mass of HCCH^+^. The counterpoise corrected binding energy was then calculated as a function of the attachment angle, which was kept frozen, while all other geometric parameters were allowed to relax. For both Ar- and Ne–HCCH^+^ the overall structure remained planar so that a symmetry plane can be distinguished (here *xz*). The label *A*′ corresponds to a symmetric and *A*′′ to an antisymmetric wavefunction with regard to this plane, corresponding to configurations (π_*x*_)^1^(π_*y*_)^2^ and (π_*x*_)^2^(π_*y*_)^1^, respectively. The resulting potential energy curves are shown in Fig. 7.

For the Ar-attachment the global minimum is found at a bent position (∼70°), where the Ar atom attaches with a binding energy of ∼1200 cm^−1^, which is in agreement with earlier computational work of Dopfer *et al.*^58^ and experimental work of Relph *et al.*^25^, where the ν_2_ C–C stretch was observed, indicating symmetry breaking effects as a result of strong Ar attachment. Furthermore, the here recorded spectrum clearly shows that the bent attachment results in a quenching of the Renner–Teller splitting, meaning that the complexation with the argon atom has lifted the degeneracy of the Π orbitals enough so that the Born–Oppenheimer approximation holds.

It is striking that the estimated binding energy (∼1200 cm^−1^) is much larger than the observed mode energy (∼720 cm^−1^). This could mean that the binding energy of the Ar is greatly overestimated by the calculations, but this seems rather unlikely due to the high level of theory used together with the Counter–Poise correction applied here. Another explanation is that the fragmentation of the Ar-complex occurs through a two-photon absorption. In order for this to happen, the difference between the *v* = 0–1 and *v* = 1–2 transitions should fall within the FELIX bandwidth. If the system would still be RT affected one would not expect this to occur, since the splitting pattern in *v* = 2 is different than in *v* = 1. However, since for the bent Ar–HCCH^+^ complex the RT splitting is quenched this difference is only determined by the anharmonicity and can be calculated with 2*ω*_5_*x*_5_ = 5.37 cm^−1^, which falls well within the FELIX bandwidth (FWHM = 5.5 cm^−1^).

**Fig. 8:**
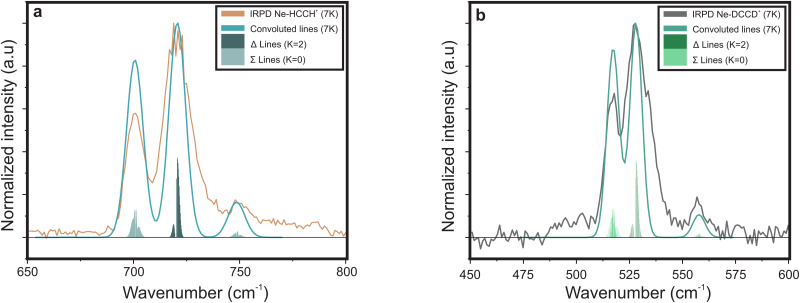
Infrared spectra of (a) Ne–HCCH^+^ and (b) Ne–DCCD^+^ simulated with the effective Hamiltonian model described in Section 2.2.2. The corresponding spectroscopic parameters are given in Table 4.

In contrast, the Ne–HCCH^+^ PES reveals two minima of similar binding energy (*D*_0_ ∼ 250 cm^−1^) at linear and bent (∼70°) geometry with a ∼40 cm^−1^ barrier. However, after correcting for the zero-point energy (see Table 3) the bent geometry is clearly favored (*D*_0_ ∼ 211 cm^−1^) compared to the linear one (*D*_0_ ∼ 96 cm^−1^). Seeing that the Renner–Teller splitting remains visible one might argue that the Ne must be attached in a linear fashion. However, the Ne atom is much more weakly bound than the Ar-atom, so that the lifting of the degeneracy of the Π orbitals in the bend minimum might not be enough to fully quench the Renner–Teller effect. To test this hypothesis we have recorded the (originally IR inactive) C–C stretch region of the Ne–HCCH^+^ complex (see ESI:† Fig. S2), but only a very weak signal could be observed (within the noise level), indeed pointing towards a weak attachment of the Ne atom. Furthermore, with a barrier of only 40 cm^−1^ between the bent and linear configuration, one might expect a large amplitude motion of the Ne sampling all geometries. It is beyond the scope of this work to calculate the behavior of the Ne-tag and its impact on the vibrational landscape *ab initio*, since it requires a full diabatic treatment including the messenger atom. However, if we assume that the Ne-atom is bound in a linear fashion we can calculate its rotational constant *ab initio*, fit the Renner–Teller parameters and make a comparison with parameters of the bare ion. To verify the used model this procedure was repeated for the Ne-tagged DCCD^+^, which shows similar behavior upon Ne tagging as HCCH^+^ (ESI:† Fig. S3). To reduce the number of fitting parameters the spin–orbit constant *A*_SO_ and anharmonic constant *x*_5_ were kept fixed. Furthermore, the temperature *T* was set to the recorded trap temperature (7 K), since no rotational substructure was visible. The resulting fits are shown in Fig. 8 and the corresponding spectroscopic parameters in Table 4.

**Table 3:** Frequencies of linear and bent Ne–HCCH^+^ complexes and the bare HCCH^+^ ion calculated at RCCSD(T)/aug-cc-pVTZ level of theory. All values are given in cm^−1^ except the dimensionless Renner–Teller constant *ε*_5_, which is given between brackets for the bending modes as (*ω*_5_, *ε*_5_)

	ν_1_	ν_2_	ν_3_	ν_4_	ν_5_	ν_6_	ν_7_	ZPE
Linear Ne–HCCH^+^	3350	3241	1839	808/579 (703, 0.321)	759/740 (750, 0.025)	92	76/72 (74, 0.054)	5779
Bent Ne–HCCH^+^	3356	3250	1840	783/558	737/727	72	20	5661
HCCH^+^	3354	3247	1839	785/558 (681, 0.329)	738/729 (734, 0.012)	…	…	5625

**Table 4:** Spectroscopic parameters used for the simulated spectra of Ne–HCCH^+^ and Ne–DCCD^+^ shown in Fig. 8. Unmarked parameters are taken from ref. 21

	*ω*_5_ (cm^−1^)	*ε*_5_	*x*_5_	*A*_SO_ (cm^−1^)	*g*_*K*_5__ (cm^−1^)	*B* (cm^−1^)	*T* (K)	FWHM (cm^−1^)
HCCH^+^ LOS	719.39(35)	−0.02737(10)	−0.00373(18)	−31.283(66)	4.545(47)	1.1028(11)	38^a^	5.5^a^
Ne–HCCH^+^ IRPD	723^a^	−0.025^a^	−0.00373(18)	−31.283(66)	6^a^	0.09^b^	7^c^	9^a^
DCCD^+^ LOS	528.5^a^	−0.02737(10)	−0.00373(18)	−31.283(66)	2.3^a^	0.80^b^	27^a^	3^a^
Ne–DCCD^+^ IRPD	531^a^	−0.024^a^	−0.00373(18)	−31.283(66)	3.2^a^	0.09^b^	7^c^	6.5^a^

a Parameters are fitted by hand.

b Parameters are calculated *ab initio* on RCCSD(T)/aug-cc-pVTZ level of theory.

c Excitation temperature was kept fixed to trap temperature.

For both the Ne–HCCH^+^ and Ne–DCCD^+^ all fitted parameters seem to change in a similar manner: the harmonic frequencies (*ω*_5_) are blue-shifted, the RT constants (*ε*_5_) decrease, the Brown parameters (*g*_*K*_5__) increase and the FWHMs increase with respect to the bare ions. The blue-shift of the harmonic frequency is in line with the *ab initio* calculations for the linear Ne–HCCH^+^ complex (see Table 3), but is much less pronounced than predicted. Furthermore, the RT parameter *ε*_5_ decreases, while an increase is predicted from the harmonic frequency calculations and the *g*_*K*_5__ increases with about one third, which is much more than would be expected if the electronic structure would be invariant to the neon attachment (then equation 
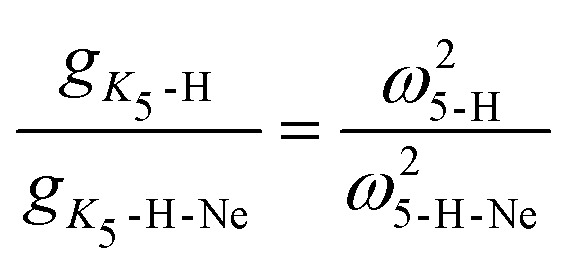
 would hold). These findings indicate that the Ne-attachment has a significant impact on the electronic landscape of Ne–HCCH^+^ and Ne–DCCD^+^.

Finally, the fitted FWHM are about a factor 2 larger than those of the bare-ion LOS and the linewidth of FELIX (3.3–6.6 cm^−1^). This cannot be fully attributed to a saturation effect, since the maximum depletion of the DCCD^+^ was only ∼30% and we correct for this in the data analysis. One could argue that the temperature of the Ne–HCCH^+^ and Ne–DCCD^+^ complexes might be significantly higher than the set trap temperature of 7 K, which may result in a broader rotational profile. To test this we have simulated the Ne–HCCH^+^ spectrum at 30 K and 50 K (see ESI:† Fig. S4). These figures clearly show that the broadness of the simulated spectra is dominated by the FWHM of the Gaussian convolution at all temperatures. The observed linewidth can therefore not be related to the rotational temperature of the complexes. Another cause of this broadening can be linked to life-time or pre-dissociation broadening effects, where a linewidth of ∼8 cm^−1^ would correspond to a very short lifetime of about 0.66 ps, which seems unlikely looking at the stability of the complex. Furthermore, it seems that the FWHM of the lines in the Δ band (highest intensity band) is larger than that of the Σ bands, since their contour is not well matched when using a universal FWHM for all lines. This could indicate that the vibronic angular momentum *K* may have an impact on the stability of the Ne-ion complex, but more likely is that the Δ band is overlayed with the rotational structure of the bent Ne–HCCH^+^ complex. The latter hypothesis is strengthened by the spectrum of the Ar–HCCH^+^ complex, which is much stronger bound than the Ne–HCCH^+^ (and should therefore have a longer lifetime), but is of similar linewidth. Of course higher-level calculations are needed to fully describe the effect of the Ne-attachment on the electronic structure, but these calculations and measurements are in line with a large amplitude motion of the Ne atom around the HCCH^+^, sampling both geometric minima.

## 4 Conclusions

In this work we have recorded the vibrational spectrum of the low-lying (∼700 cm^−1^) *cis*-bending of HCCH^+^ with the novel spectroscopic method leak-out spectroscopy (LOS),^28^ acting as a proof-of-principle for the application of this method to low-energy vibrational modes. The effective Hamiltonian fit allowed to directly compare our work to the spectrum previously recorded at 90 K by means of LIR^23,24^, allowing to attribute the differences between the spectra to the ion temperature. Finally, this work provided some much-needed clarification regarding the large discrepancies between the spectroscopic parameters of the RT-perturbed *cis*-bending mode of HCCH^+^ reported in different experimental works. The excellent agreement between our work and the parameters derived from PFI-ZEKE PES by Tang *et al.*^21^ leads us to believe that their data-set provides the most accurate description of the HCCH^+^ ion. Extending on these measurements we have recorded the first vibrational spectrum of the *cis*-bending mode of DCCD^+^ and the corresponding spectroscopic parameters were extracted with the same model as used for HCCH^+^. The obtained parameters may be used for future benchmarking studies related to isotope substitution in RT affected species.

In addition, we have recorded the vibrational spectra of HCCH^+^ and DCCD^+^ by means of the commonly used infrared predissociation (IRPD) action spectroscopic method, using Ne and Ar as messenger atoms. We found that the Ne-attachment leads to a distortion of the RT splitting and Ar to a complete quenching, which is supported by both *ab initio* calculations and an effective Hamiltonian analysis. The IRPD method is often used under the assumption that the spectrum of the weakly-bound complex can be taken as proxy for that of the bare ion. Seeing that this is clearly not the case for HCCH^+^ and DCCD^+^ and that similar effects were reported for HC_3_N^+^,^42^ where it was found that the Ne attachment affected the RT splitting, we argue that spectra obtained with the IRPD method should not be taken as a proxy for the bare ion spectra for RT affected species. In these cases LOS provides an excellent universal alternative.

In contrast to most closed-shell species, the state of *ab initio* calculations for the simulation of the ro-vibrational spectra of Renner–Teller perturbed molecules is relatively poor, as shown here for DCCD^+^. It is therefore particularly important to obtain accurate experimental spectroscopic constants that are used to simulate the radio- or infrared spectra through which these species are detected. The newly launched James–Webb Space Telescope (JWST) provides mid-resolution observational infrared data at unprecedented sensitivity, and a means of detection for low-abundance symmetric species with no rotational spectrum like HCCH^+^. The spectroscopic parameters now derived and confirmed for the highly characteristic *cis*-bendings of DCCD^+^ and HCCH^+^ may be used for follow-up high-resolution studies or may even be sufficient for direct comparison to JWST data, as has recently been demonstrated for the hydrocarbon ion CH_3_^+^.^59,60^ This paper also highlights the complementary nature of PFI-ZEKE PES and infrared LOS, where PFI-ZEKE may provide rotationally resolved spectra and accurate spectroscopic constants and LOS information on expected line intensities and selection rules. The demonstration of LOS as a universal broadband infrared action spectroscopic method as shown here, opens the door for future studies of other astro- and fundamentally relevant RT affected cations, such as linear HCCCH^+^, CCH^+^ and HCCN^+^.

## Author contributions

K. S.: conceptualization, data curation, formal analysis, investigation, methodology, software, visualization, writing-original draft. T. V. B.: data curation, formal analysis, investigation. G. C. G.: methodology, software, supervision, validation, writing – review & editing. O. A.: conceptualization, validation, writing – review& editing. S. S: conceptualization, validation, writing – review& editing. B. R.: funding acquisition, resources, supervision, writing – review & editing. S. B.: conceptualization, data curation, funding acquisition, investigation, methodology, project administration, resources, supervision, writing – review & editing.

## Conflicts of interest

There are no conflicts to declare.

## Supplementary Material

CP-026-D3CP04989B-s001
