# Supplementary material for: Leak-out spectroscopy as alternative method to rare-gas tagging for the Renner–Teller perturbed HCCH+ and DCCD+ ions
Source: Phys Chem Chem Phys. 2023 Dec 19;26(3):2692–703. doi: 10.1039/d3cp04989b (PMC10793980; doi:10.1039/d3cp04989b)
Supplement: CP-026-D3CP04989B-s001 [file CP-026-D3CP04989B-s001.pdf]

Electronic Supplementary Material (ESI) for Physical Chemistry Chemical Physics.
This journal is © the Owner Societies 2023

Supplementary Material: Leak-out spectroscopy as alternative method to rare-gas tagging for the Renner-Teller perturbed $HCCH^+$ and $DCCD^+$ ions

Kim Steenbakkers,[1] Tom van Boxtel,[1] Gerrit C. Groenenboom,[2] Oskar Asvany,[3] Britta Redlich,[1] Stephan Schlemmer,[3] and Sandra Brünken[1, a)]

[1)] *Radboud University, FELIX Laboratory, Institute for Molecules and Materials, Nijmegen, The Netherlands*

[2)] *Radboud University, Institute for Molecules and Materials, Nijmegen, The Netherlands*

[3)] *Physikalisches Institut der Universität zu Köln, Köln, Germany*

[a)] Author to whom correspondence should be addressed: sandra.bruenken@ru.nl

I. LINE STRENGTH DERIVATION

A. Ro-vibronic wave functions

We use the shorthand notation for the vibronic basis functions defined in the Sec. 2.2.2 of the main text

$$|\boldsymbol{n}\rangle = |\Lambda\rangle|S\Sigma\rangle|v_5 l_5\rangle|K\rangle, \tag{1}$$

where $K = \Lambda + l_5$ is a redundant quantum number. The projection quantum numbers refer to the molecular axis with spherical polar angles (θ, ϕ), which we take to be the z-axis of a two-angles embedded molecular frame

$$\boldsymbol{R}(\phi, \theta, 0) = \boldsymbol{R}_z(\phi)\boldsymbol{R}_y(\theta), \tag{2}$$

where $\boldsymbol{R}_z(\phi)$ is the 3×3 matrix representing rotation around the z-axis over the angle ϕ, and $\boldsymbol{R}_y(\theta)$ is the rotation around the y-axis. The two-angle embedded parity-unadapted rovibronic basis functions are

$$|\boldsymbol{n}PJM\rangle = D^{J,*}_{M,P}(\phi, \theta, 0)\hat{R}(\phi, \theta, 0)|\boldsymbol{n}\rangle^{\text{SF}}\sqrt{\frac{[J]}{4\pi}}, \tag{3}$$

where the rotational part of the wave function is given by a Wigner D-matrix element in the zyz-Euler angle parametrization in the active convention[1] and $P = K + \Sigma$. The superscript SF on the vibronic wave function indicates that the projection quantum numbers refer to the space fixed axis and

$$\hat{R}(\phi, \theta, 0) = \hat{R}_z(\phi)\hat{R}_y(\theta) \tag{4}$$

is a rotation operator which rotates the space-fixed functions to the two-angle embedded molecular frame,

$$|\boldsymbol{n}\rangle = \hat{R}(\phi, \theta, 0)|\boldsymbol{n}\rangle^{\text{SF}}. \tag{5}$$

This rotation operator formulation is described in the appendix of Ref.[2], where it is also shown how to evaluate the effect of the space inversion operator $\hat{i}$, which is required for parity adaption:

$$\begin{aligned}\hat{i}|\boldsymbol{n}PJM\rangle =& (-1)^{J-P}D^{J,*}_{M,-P}(\phi, \theta, 0)\sqrt{\frac{[J]}{4\pi}} \\ & \times \hat{R}(\phi, \theta, 0)\,\hat{R}_y(\pi)\hat{i}|\boldsymbol{n}\rangle^{\text{SF}}.\end{aligned} \tag{6}$$

For spatial coordinates the product of the rotation around the y-axis times inversion is a reflection in the xz-plane

$$\hat{R}_y(\pi)\hat{i} = \hat{\sigma}_v(xz). \tag{7}$$

For the electronic wave function we use the convention

$$\hat{\sigma}_v(xz)|\Lambda\rangle = (-1)^{\Lambda}|-\Lambda\rangle. \tag{8}$$

For the bend-vibrational functions defined in the next section we have

$$\hat{\sigma}_v(xz)|v_5 l_5\rangle = |v_5, -l_5\rangle. \tag{9}$$

The space-fixed electron spin function is invariant under inversion. The rotation around the y-axis gives

$$\hat{R}_y(\pi)|S\Sigma\rangle^{\mathrm{SF}} = (-1)^{S-\Sigma}|S, -\Sigma\rangle. \tag{10}$$

Putting everything together, including the redundant quantum numbers, we find

$$\begin{aligned} \hat{i}|\Lambda S\Sigma v_5 l_5 K; PJM\rangle = (-1)^{J-S+l_5} \\ |-\Lambda, S, -\Sigma, v_5, -l_5, -K; -P, J, M\rangle. \end{aligned} \tag{11}$$

Parity adapted functions with $p = \pm 1$ are defined by

$$|\boldsymbol{n}PJMp\rangle = \frac{1 + p\hat{i}}{\sqrt{2}}|\boldsymbol{n}PJM\rangle. \tag{12}$$

The normalization factor is $1/\sqrt{2}$, since we have $\Lambda \neq 0$ (and also $\Sigma \neq 0$) and hence none of the functions $|\boldsymbol{n}PJM\rangle$ is invariant under inversion. In the parity adapted basis we take $\Lambda = 1$.

We expand the wave function as

$$|\Psi_i^{JMp}\rangle = \sum_{\boldsymbol{n}} |\boldsymbol{n}JMp\rangle u_{\boldsymbol{n},i}^{(Jp)}. \tag{13}$$

B. Line strength

The line strength is defined by

$$S(J'p'i'; Jpi) = \sum_{M'mM} |\langle\Psi_{i'}^{J'M'p'}|\hat{\mu}_{5,m}^{\mathrm{SF}}|\Psi_i^{JMp}\rangle|^2, \tag{14}$$

where the spherical components $\hat{\mu}_m^{\mathrm{SF}}$ of the space-fixed dipole operator are related to the Cartesian components through

$$\hat{\mu}_{5,\pm 1}^{\mathrm{SF}} = \mp \frac{\hat{\mu}_{5,x}^{\mathrm{SF}} \pm i\hat{\mu}_{5,y}^{\mathrm{SF}}}{\sqrt{2}} \tag{15}$$

$$\hat{\mu}_{5,0}^{\mathrm{SF}} = \hat{\mu}_{5,z}^{\mathrm{SF}}. \tag{16}$$

To evaluate the dipole operator matrix elements for the parity unadapted basis,

$$\mu_{5,m} = \langle \boldsymbol{n}'P'J'M'|\hat{\mu}^{\mathrm{SF}}_{5,m}|\boldsymbol{n}PJM\rangle \tag{17}$$

we use

$$\hat{R}^{\dagger}\hat{\mu}^{\mathrm{SF}}_{5,m}\hat{R} = \sum_{k=1}^{1} \hat{\mu}^{\mathrm{SF}}_{5,k} D^{1,*}_{mk}(\phi,\theta,0), \tag{18}$$

which gives

$$\mu_{5,m} = \sum_{k=-1}^{1} \langle \boldsymbol{n}'|\hat{\mu}_{5,k}|\boldsymbol{n}\rangle \frac{\sqrt{[J',J]}}{4\pi}\int_{-1}^{1} d\cos\theta \int_{0}^{2\pi} d\phi$$
$$\times D^{(J')}_{M'P'}(\phi,\theta,0) D^{1,*}_{mk}(\phi,\theta,0) D^{J,*}_{MP}(\phi,\theta,0). \tag{19}$$

The dipole matrix element is zero unless

$$P' = k + P, \tag{20}$$

so we may drop the summation and take $k = P' - P$. Also, this allows us to introduce a third Euler angle γ in the integration by inserting

$$1 = \frac{1}{2\pi}\int_{0}^{2\pi} e^{i(-P'+k+P)\gamma} d\gamma, \tag{21}$$

so we can use integrate the product of Wigner D-matrices analytically,

$$\mu_{5,m} = [J',J]^{\frac{1}{2}} \langle \boldsymbol{n}'|\hat{\mu}_{5,k}|\boldsymbol{n}\rangle$$
$$\times \begin{pmatrix} J' & 1 & J \\ \bar{M}' & m & M \end{pmatrix} \begin{pmatrix} J' & 1 & J \\ \bar{P}' & k & P \end{pmatrix}. \tag{22}$$

Here, the bar over the projection quantum numbers M' and P' in the 3-j symbol indicate "time reversal", i.e., it flips the sign of the projection quantum number and includes a phase factor, e.g.,

$$\begin{pmatrix} J' & 1 & J \\ \bar{M}' & m & M \end{pmatrix} = (-1)^{J'-M'} \begin{pmatrix} J' & 1 & J \\ -M' & m & M \end{pmatrix}. \tag{23}$$

The dipole operator changes sign under inversion

$$\hat{\imath}\hat{\mu}^{\mathrm{SF}}_{5,m} = -\hat{\mu}^{\mathrm{SF}}_{5,m}\hat{\imath} \tag{24}$$

so matrix elements are only nonzero for states with opposite parity, $p' = -p$, so when evaluating dipole matrix elements in the parity adapted basis we may use

$$\frac{1+p'\hat{i}}{\sqrt{2}}\hat{\mu}^{\mathrm{SF}}_{5,m}\frac{1+p\hat{i}}{\sqrt{2}} = \hat{\mu}^{\mathrm{SF}}_{5,m} + p\hat{\mu}^{\mathrm{SF}}_{5,m}\hat{i}. \tag{25}$$

In our approximation of the dipole operator discussed in section I E, matrix elements of the second term are zero, so we have

$$\langle \boldsymbol{n}'P'J'M'p'|\hat{\mu}^{\mathrm{SF}}_{5,m}|\boldsymbol{n}PJMp\rangle = \\ \delta_{p',-p}\langle \boldsymbol{n}'P'J'M'|\hat{\mu}^{\mathrm{SF}}_{5,m}|\boldsymbol{n}PJM\rangle. \tag{26}$$

Substituting the expansion of the wave function [Eq. (13)] into the expression for the line strength [Eq. (14)], using Eq. (22) and

$$\sum_{M'mM}\left|\begin{pmatrix} J' & 1 & J \\ \bar{M}' & m & M \end{pmatrix}\right|^2 = 1, \tag{27}$$

we find for the line strength

$$S(J'p'i'lJpi) = (2J'+1)(2J+1) \\ \times\left|\sum_{\boldsymbol{n}'\boldsymbol{n}}\langle \boldsymbol{n}'|\hat{\mu}_{5,k}|\boldsymbol{n}\rangle\begin{pmatrix} J' & 1 & J \\ \bar{P}' & k & P \end{pmatrix} u^{(J'p')*}_{\boldsymbol{n}',i'}u^{(Jp)}_{\boldsymbol{n},i}\right|^2. \tag{28}$$

C. Cis-bending mode

The degenerate ν_5 cis-bending normal mode of $HCCH^+$ follows from the symmetry of the system. We define the Cartesian coordinates of the atoms with respect to a molecule-fixed frame $\boldsymbol{R}$, such that in the equilibrium structure the molecule is along the z-axis. In this frame the x-coordinates of the hydrogen atoms are the same and we denote them by x_{H}. The x-coordinates of the carbon atoms, x_{C}, are also the same. We define the normal coordinate $q_{5,x}$ for bending in the xz-plane such that

$$q_{5,x} = x_{\mathrm{H}} - x_{\mathrm{C}}, \tag{29}$$

The origin of the coordinate system is the center-of-mass of the system, so we have

$$m_{\mathrm{H}}x_{\mathrm{H}} + m_{\mathrm{C}}x_{\mathrm{C}} = 0, \tag{30}$$

where m_{H} and m_{C} are the atomic masses for H and C, respectively. The Cartesian coordinates as function of the normal coordinates $q_{5,x}$ are

$$x_{\mathrm{H}} = \frac{m_{\mathrm{C}}}{m_{\mathrm{C}} + m_{\mathrm{H}}} q_{5,x} \tag{31}$$

$$x_{\mathrm{C}} = -\frac{m_{\mathrm{H}}}{m_{\mathrm{C}}} \frac{m_{\mathrm{C}}}{m_{\mathrm{C}} + m_{\mathrm{H}}} q_{5,x}. \tag{32}$$

The Hamiltonian for vibration in the xz-plane in this coordinate in the harmonic approximation is

$$\hat{H}_{5,x} = -\frac{\hbar^2}{2\mu_5} \frac{\partial^2}{\partial q_{5,x}^2} + \frac{1}{2} k_5 q_{5,x}^2, \tag{33}$$

where the reduced mass is given by

$$\mu_5 = 2 \frac{m_{\mathrm{C}} m_{\mathrm{H}}}{m_{\mathrm{C}} + m_{\mathrm{H}}}. \tag{34}$$

The harmonic force constant k_5 is related to the harmonic frequency through

$$\omega_5 = \sqrt{\frac{k_5}{\mu_5}}. \tag{35}$$

We introduce the dimensionless normal coordinate

$$\hat{Q}_{5,x} = q_{5,x} \sqrt{\frac{\mu_5 \omega_5}{\hbar}} \tag{36}$$

such that the Hamiltonian becomes

$$\hat{H}_{5,x} = \frac{1}{2} \hbar \omega (\hat{P}_{5,x}^2 + \hat{Q}_{5,x}^2), \tag{37}$$

where $\hat{P}_{5,x}$ is the momentum conjugate to $\hat{Q}_{5,x}$ and the commutator is

$$[\hat{P}_{5,x}, \hat{Q}_{x,5}] = 1. \tag{38}$$

The annihilation operator $\hat{a}_{5,x}$ is defined by

$$\hat{a}_{5,x} = \frac{1}{\sqrt{2}} (\hat{Q}_{5,x} + i\hat{P}_{5,x}) \tag{39}$$

and the creation operator $\hat{a}_{5,x}^{\dagger}$ is its Hermitian conjugate. With the analogous definitions for bending in the yz-plane, the two-dimensional harmonic oscillator Hamiltonian for the ν_5 bending mode is given by

$$\hat{H} = \hat{H}_x + \hat{H}_y = \hbar \omega_5 (\hat{N} + 1), \tag{40}$$

where the number operator

$$\hat{N} = \hat{N}_{5,x} + \hat{N}_{5,y} \tag{41}$$

with the number operators for $v_{5,x}$ and $v_{5,y}$ defined by

$$\hat{N}_x = \hat{a}^\dagger_{5,x}\hat{a}_{5,x} \tag{42}$$

$$\hat{N}_y = \hat{a}^\dagger_{5,y}\hat{a}_{5,y}. \tag{43}$$

The vibrational angular momentum operator is given by

$$\hat{L}_5 = \hat{Q}_{5,x}\hat{P}_{5,y} - \hat{Q}_{5,y}\hat{P}_{5,x}. \tag{44}$$

Working with vibrational wave functions with well-defined vibrational angular momentum is particularly convenient for Renner-Teller problems, so we switch to complex coordinates and their conjugate momenta,

$$\hat{Q}_{5,\pm} = \frac{1}{\sqrt{2}}(\hat{Q}_{5,x} \pm i\hat{Q}_{5,y}) \tag{45}$$

$$\hat{P}_{5,\pm} = \frac{1}{\sqrt{2}}(\hat{P}_{5,x} \pm i\hat{P}_{5,y}). \tag{46}$$

This constitutes a unitary transformation of the coordinates so the commutation relations are invariant, i.e.,

$$[\hat{P}_{5,\pm}, \hat{Q}_{5,\pm}] = 1, \tag{47}$$

while all other commutators of these complex operators are zero. The corresponding creation operators are given by

$$\hat{a}^\dagger_{5,\pm} = \frac{1}{\sqrt{2}}(\hat{a}^\dagger_{5,x} \pm i\hat{a}^\dagger_{5,y}) \tag{48}$$

$$\hat{a}_{5,\pm} = \frac{1}{\sqrt{2}}(\hat{a}_{5,x} \mp i\hat{a}_{5,y}). \tag{49}$$

The commutators of these creation and annihilation operators are all zero, except for

$$[\hat{a}_{5,\pm}, \hat{a}^\dagger_{5,\pm}] = 1. \tag{50}$$

The complex coordinates can be expressed in these operators as

$$\hat{Q}_{5,\pm} = \hat{a}_{5,\mp} + \hat{a}^\dagger_{5,\pm} \tag{51}$$

and number and vibrational angular momentum operators can now be written as

$$\hat{N}_5 = \hat{N}_{5,+} + \hat{N}_{5,-} \tag{52}$$

$$\hat{L}_5 = \hat{N}_{5,+} - \hat{N}_{5,-} \tag{53}$$

with

$$\hat{N}_{5,\pm} = \hat{a}^\dagger_{5,\pm}\hat{a}_{5,\pm}. \tag{54}$$

These operators commute, $[\hat{N}_{5,+}, \hat{N}_{5,-}] = 0$, so we can define two-dimensional harmonic oscillator eigenfunctions

$$|n_{5,+}n_{5,-}\rangle^{+,-} = \frac{1}{\sqrt{n_{5,+}!n_{5,-}!}}(\hat{a}^\dagger_{5,+})^{n_{5,+}}(\hat{a}^\dagger_{5,-})^{n_{5,-}}|0,0\rangle, \tag{55}$$

where the normalized, two-dimensional harmonic oscillator ground state in canonical coordinates is given by

$$\langle Q_{5,x}, Q_{5,y}|0,0\rangle = \sqrt{\frac{\mu_5\omega_5}{\pi\hbar}}\exp\left(-\frac{\mu_5\omega_5}{2\hbar}Q_5^2\right), \tag{56}$$

with $Q_5^2 = Q_{5,x}^2 + Q_{5,y}^2$. For these $+,-$ eigenfunctions we have

$$\hat{N}_5|n_{5,+}n_{5,-}\rangle^{+,-} = (n_{5,+} + n_{5,-})|n_{5,+}n_{5,-}\rangle^{+,-} \tag{57}$$

$$\hat{L}_5|n_{5,+}n_{5,-}\rangle^{+,-} = (n_{5,+} - n_{5,-})|n_{5,+}n_{5,-}\rangle^{+,-}. \tag{58}$$

Hence, we can define the $|v_5 l_5\rangle$ basis functions, with quantum numbers

$$v_5 = n_{5,+} + n_{5,-} \tag{59}$$

$$l_5 = n_{5,+} - n_{5,-} \tag{60}$$

by

$$|v_5 l_5\rangle = |\frac{v_5 + l_v}{2}, \frac{v_5 - l_5}{2}\rangle^{+,-}, \tag{61}$$

so that we have

$$\hat{N}_5|v_5 l_5\rangle = v_5|v_5 l_5\rangle \tag{62}$$

$$\hat{L}_5|v_5 l_5\rangle = l_5|v_5 l_5\rangle. \tag{63}$$

The action of the ladder operators follow from

$$\hat{a}^\dagger_{5,+}|n_{5,+}n_{5,-}\rangle^{+,-} = \sqrt{n_{5,+}+1}|n_{5,+}+1, n_{5,-}\rangle^{+,-} \tag{64}$$

$$\hat{a}_{5,+}|n_{5,+}n_{5,-}\rangle^{+,-} = \sqrt{n_{5,+}}|n_{5,+}-1, n_{5,-}\rangle^{+,-} \tag{65}$$

and $\hat{a}_{5,-}$ its Hermitian conjugate act on $n_{5,-}$ analogously. Finally we can evaluate the matrix elements of Eq. (75). For example, for $k=1$, $l_5' = l_5+1$, and $v_5' = v_5+1$ we find

$$\begin{aligned}
&\langle v_5+1, l_5+1|\hat{Q}_{5,+}|v_5, l_5\rangle \\
&\quad = \langle v_5+1, l_5+1|\hat{a}_{5,-} + \hat{a}^\dagger_{5,+}|v_5, l_5\rangle && (66)\\
&\quad = \langle v_5+1, l_5+1|\hat{a}^\dagger_{5,+}|v_5, l_5\rangle && (67)\\
&\quad = \sqrt{n_{5,+}+1} = \sqrt{\frac{v_5+l_5}{2}+1}. && (68)
\end{aligned}$$

These matrix elements agree with the expressions in Appendix E.2 in Ref.[3].

D. Reflection symmetry

Reflection in the molecule-fixed xz coordinates gives

$$\hat{\sigma}_v(xz)\hat{Q}_{5,x}\hat{\sigma}^\dagger_v(xz) = \hat{Q}_{5,x} \quad (69)$$

$$\hat{\sigma}_v(xz)\hat{Q}_{5,y}\hat{\sigma}^\dagger_v(xz) = -\hat{Q}_{5,y}. \quad (70)$$

For the creation operators this gives

$$\hat{\sigma}_v(xz)\hat{a}^\dagger_{5,\pm}\hat{\sigma}^\dagger_v(xz) = \hat{a}^\dagger_{5,\mp}. \quad (71)$$

Applying the reflection operators to the $+,-$ basis [Eq. (55)] gives

$$\hat{\sigma}_v(xz)|n_{5,+}n_{5,-}\rangle^{+,-} = |n_{5,-}n_{5,+}\rangle^{+,-}, \quad (72)$$

so we also have

$$\hat{\sigma}_v(xz)|v_5 l_5\rangle = |v_5, -l_5\rangle. \quad (73)$$

E. Dipole matrix elements

We approximate the dipole operator by only including terms linear in the normal coordinate,

$$\hat{\mu}_{5,\pm1} = \mp\mu_5^\perp \hat{Q}_{5,\pm} \quad (74)$$

, where $\mu_5^\perp$ is the perpendicular dipole moment, and we neglect contributions nondiagonal in Λ. The dipole operator does not act on the electron spin, so we have

$$\langle \boldsymbol{n}'|\hat{\mu}_{5,k}|\boldsymbol{n}\rangle = \mp\mu_5^\perp \delta_{\Sigma'\Sigma}\delta_{\Lambda'\Lambda}\langle v_5' l_5'|\hat{Q}_{5,k}|v_5 l_5\rangle, \quad (75)$$

where the matrix elements of the normal coordinate are given in the previous section.

In our basis $\Lambda = 1$ and our approximate dipole operator does not act on the electronic coordinate Λ we dropped the second term in Eq. (25).

II. TABLES AND FIGURES

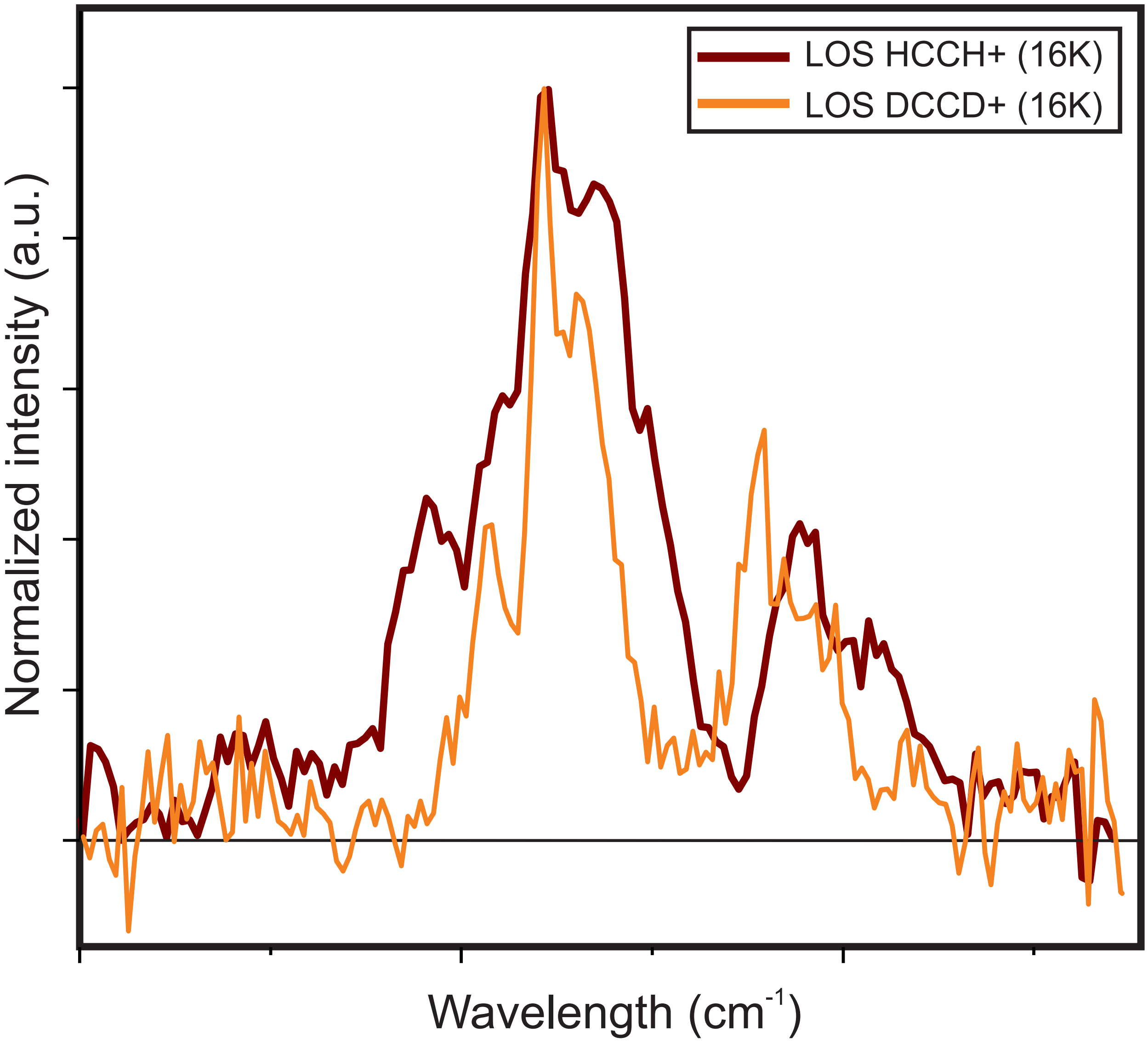

FIG. 1: Overlayed and scaled $DCCD^+$ and $HCCH^+$ spectra recorded by means of LOS

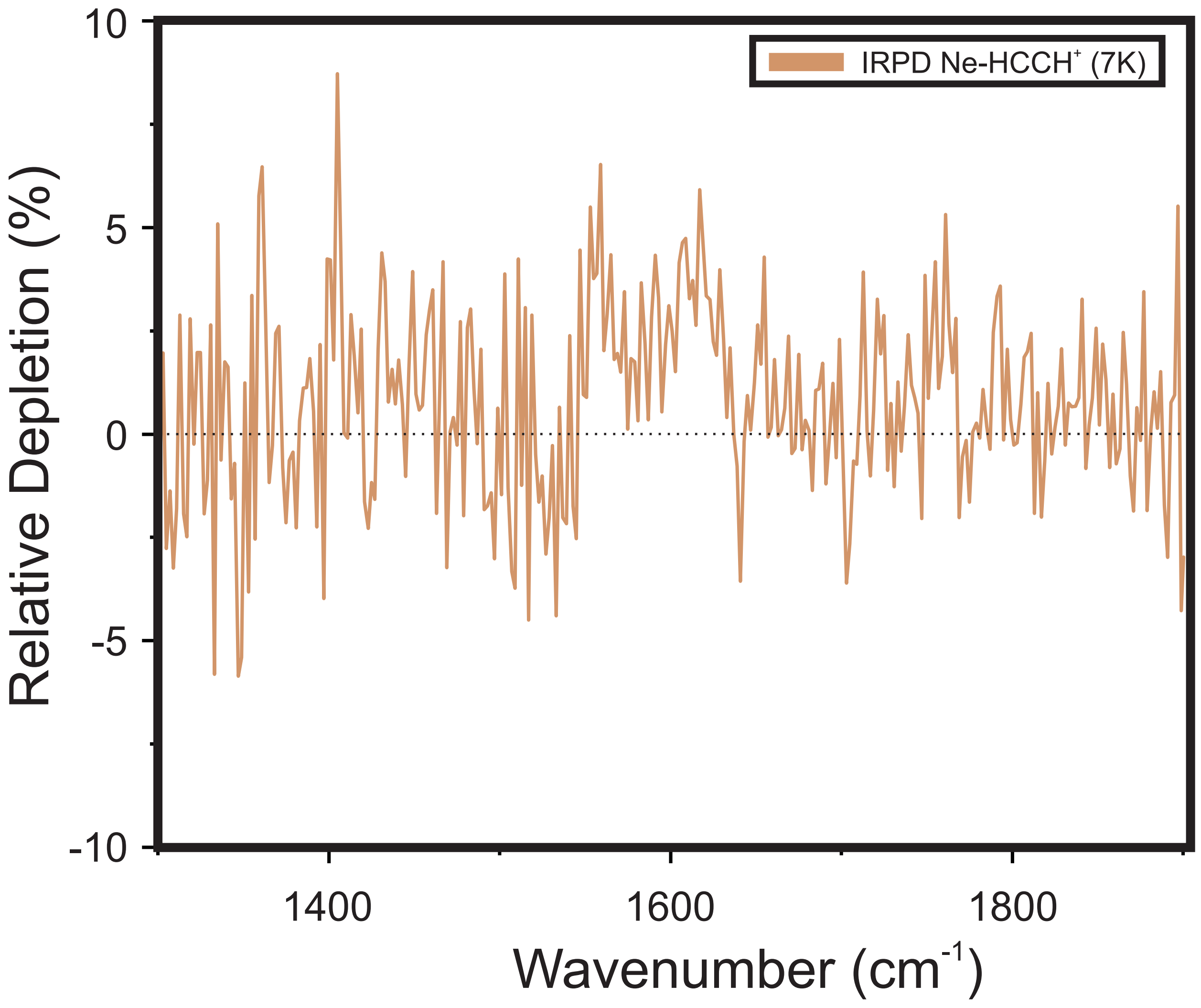

FIG. 2: Vibrational IRPD spectrum of the Ne−$HCCH^+$ recorded in the C-C stretching region.

TABLE I: Calculated frequencies and intensities based on fitted spectroscopic parameters of[4] of the Sigma states

Freq (cm^{-1})	Int. 38K (a.u.)	Int. 90K (a.u.)	J'	J"
639.8	0.0023	0.010	6.5	7.5
643.4	0.0035	0.011	5.5	6.5
647.0	0.0047	0.011	4.5	5.5
650.7	6.4E-4	0.012	10.5	10.5

continued on next page

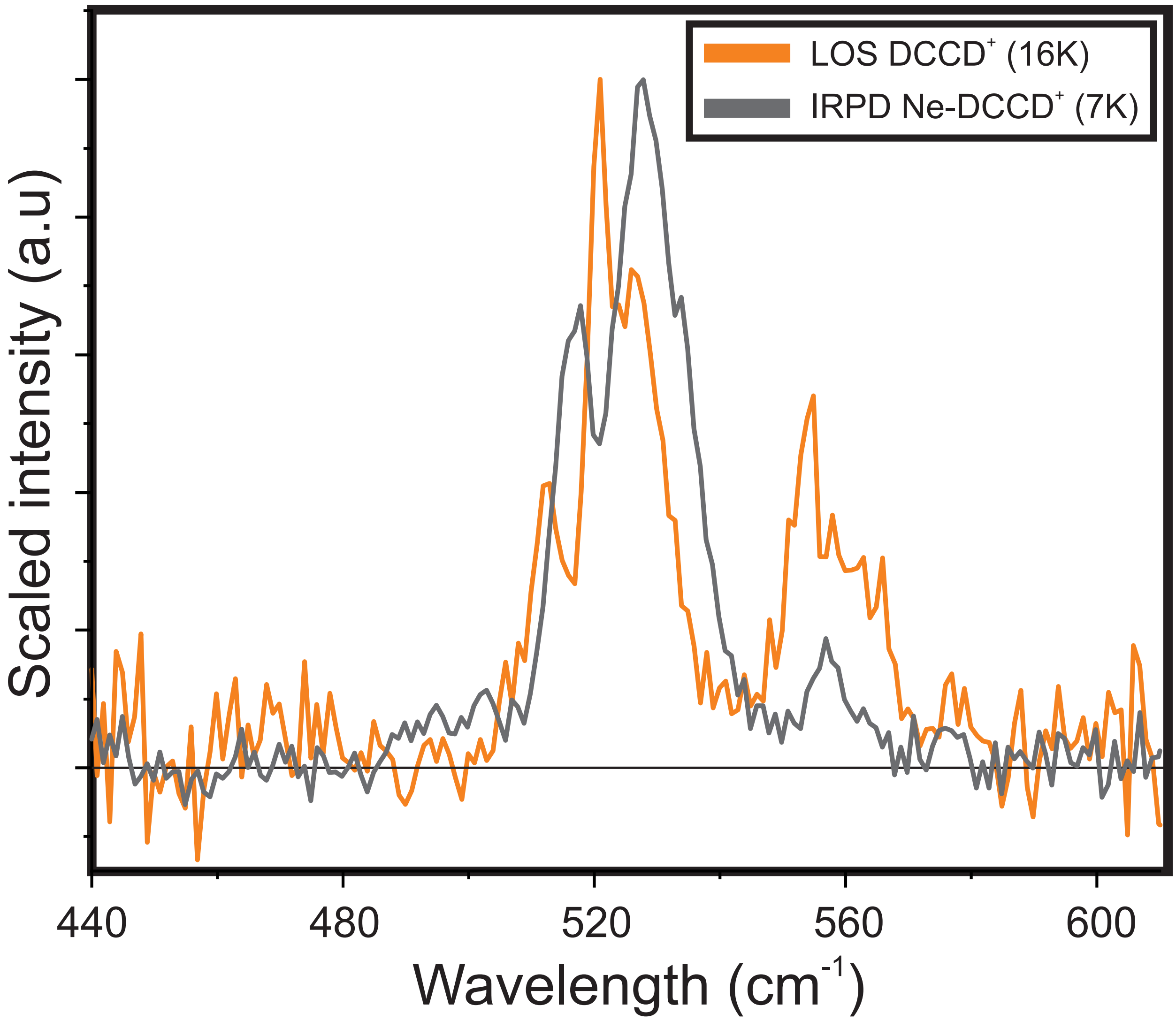

FIG. 3: Comparison of the infrared spectra of the bare $DCCD^+$ ion taken with LOS and of Ne-$DCCD^+$ recorded by means of IRPD.

TABLE I – continued from previous page

Freq (cm^{-1})	Int. 38K (a.u.)	Int. 90K (a.u.)	J'	J"
652.3	0.0014	0.015	9.5	9.5
653.9	0.0026	0.019	8.5	8.5
655.4	0.0046	0.021	7.5	7.5
656.8	0.0071	0.022	6.5	6.5

continued on next page

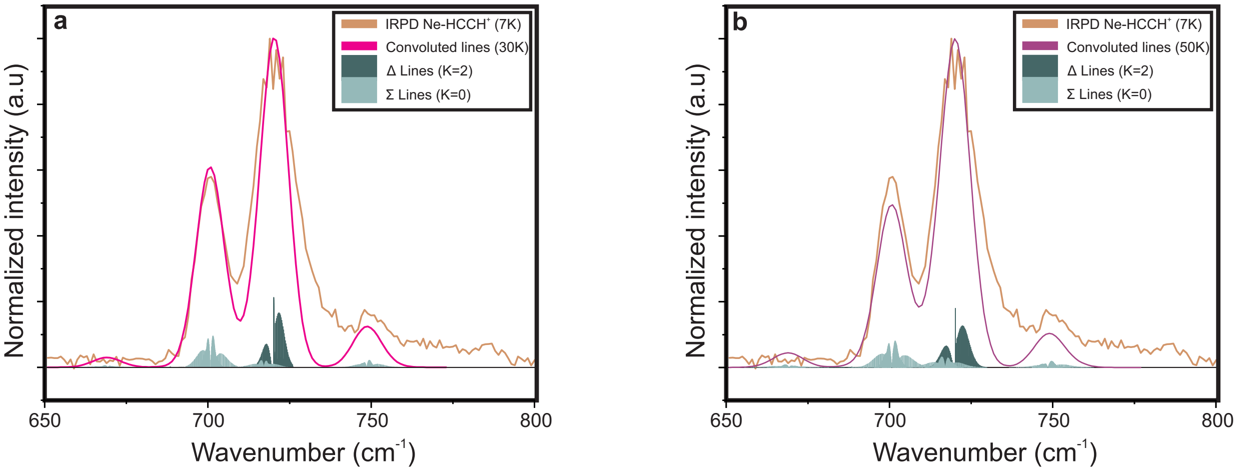

FIG. 4: Infrared spectrum of Ne-$HCCH^+$ overlayed with the theoretical spectra simulated at 30K (a) and 50K (b).

TABLE I – continued from previous page

Freq (cm^{-1})	Int. 38K (a.u.)	Int. 90K (a.u.)	J'	J"
658.2	0.0098	0.022	5.5	5.5
659.5	0.012	0.021	4.5	4.5
660.8	0.013	0.018	3.5	3.5
662.0	0.011	0.013	2.5	2.5
670.7	0.0061	0.011	5.5	4.5
671.6	0.0050	0.011	6.5	5.5
672.4	0.0030	0.014	8.5	9.5
672.4	0.0036	0.011	7.5	6.5
673.2	0.0023	0.011	8.5	7.5
675.0	0.0063	0.019	7.5	8.5
677.6	0.012	0.025	6.5	7.5
680.3	0.022	0.031	5.5	6.5
683.0	0.034	0.036	4.5	5.5
684.3	0.0011	0.013	8.5	9.5
684.3	4.7E-4	0.011	11.5	12.5

continued on next page

TABLE I – continued from previous page

Freq (cm^{-1})	Int. 38K (a.u.)	Int. 90K (a.u.)	J'	J"
685.4	0.0012	0.016	10.5	11.5
685.7	0.050	0.041	3.5	4.5
686.4	0.0029	0.022	9.5	10.5
687.4	0.0063	0.030	8.5	9.5
687.7	0.0023	0.016	7.5	8.5
688.4	0.012	0.037	7.5	8.5
688.5	0.065	0.043	2.5	3.5
689.4	0.022	0.045	6.5	7.5
690.5	0.036	0.051	5.5	6.5
691.1	0.0042	0.019	6.5	7.5
691.4	0.077	0.043	1.5	2.5
691.5	0.053	0.056	4.5	5.5
691.7	8.5E-4	0.011	11.5	11.5
692.0	0.0021	0.016	10.5	10.5
692.3	0.0048	0.023	9.5	9.5
692.6	0.071	0.058	3.5	4.5
692.7	0.010	0.030	8.5	8.5
693.2	0.019	0.038	7.5	7.5
693.6	0.033	0.046	6.5	6.5
693.7	0.085	0.056	2.5	3.5
694.2	0.050	0.053	5.5	5.5
694.3	0.083	0.041	0.5	1.5
694.4	0.0068	0.022	5.5	6.5
694.7	0.069	0.056	4.5	4.5
694.9	0.092	0.052	1.5	2.5
695.3	0.083	0.054	3.5	3.5

continued on next page

TABLE I – continued from previous page

Freq (cm^{-1})	Int. 38K (a.u.)	Int. 90K (a.u.)	J’	J”
696.0	0.084	0.047	2.5	2.5
696.0	0.091	0.045	0.5	1.5
696.7	0.063	0.031	1.5	1.5
697.8	0.0099	0.023	4.5	5.5
700.2	0.076	0.038	1.5	1.5
701.1	0.013	0.022	3.5	4.5
701.2	0.11	0.061	2.5	2.5
702.2	0.12	0.078	3.5	3.5
702.4	4.2E-4	0.014	11.5	11.5
702.8	0.025	0.014	3.5	2.5
703.3	0.11	0.087	4.5	4.5
703.5	0.0010	0.020	10.5	10.5
704.3	0.027	0.018	4.5	3.5
704.4	0.085	0.089	5.5	5.5
704.4	0.014	0.020	2.5	3.5
704.7	0.0024	0.027	9.5	9.5
704.7	0.0036	0.012	5.5	6.5
705.5	0.059	0.085	6.5	6.5
705.8	0.0048	0.034	8.5	8.5
705.9	0.024	0.020	5.5	4.5
706.4	0.0057	0.013	4.5	5.5
706.6	0.037	0.076	7.5	7.5
706.9	0.0088	0.041	7.5	7.5
707.5	0.019	0.020	6.5	5.5
707.7	0.013	0.015	1.5	2.5
707.8	0.021	0.064	8.5	8.5

continued on next page

TABLE I – continued from previous page

Freq (cm^{-1})	Int. 38K (a.u.)	Int. 90K (a.u.)	J'	J"
708.0	0.0080	0.014	3.5	4.5
708.1	0.015	0.046	6.5	6.5
708.9	0.011	0.051	9.5	9.5
709.2	0.022	0.050	5.5	5.5
709.2	0.012	0.018	7.5	6.5
709.6	0.0096	0.013	2.5	3.5
709.7	0.036	0.020	3.5	2.5
710.1	0.0051	0.039	10.5	10.5
710.3	0.028	0.050	4.5	4.5
710.9	0.0075	0.015	8.5	7.5
711.2	0.0097	0.011	1.5	2.5
711.2	0.0022	0.029	11.5	11.5
711.4	0.033	0.046	3.5	3.5
712.3	8.3E-4	0.020	12.5	12.5
712.4	0.033	0.039	2.5	2.5
712.7	0.0040	0.012	9.5	8.5
712.9	0.043	0.028	4.5	3.5
713.3	2.9E-4	0.013	13.5	13.5
713.4	0.027	0.028	1.5	1.5
714.3	0.018	0.018	0.5	0.5
716.1	0.018	0.017	0.5	0.5
716.1	0.041	0.034	5.5	4.5
716.9	0.023	0.024	1.5	1.5
717.6	0.026	0.030	2.5	2.5
718.1	0.015	0.016	2.5	1.5
718.2	0.023	0.033	3.5	3.5

continued on next page

TABLE I – continued from previous page

Freq (cm^{-1})	Int. 38K (a.u.)	Int. 90K (a.u.)	J'	J"
718.9	0.019	0.033	4.5	4.5
719.3	0.018	0.021	3.5	2.5
719.4	0.034	0.036	6.5	5.5
719.4	0.013	0.030	5.5	5.5
720.0	0.0081	0.026	6.5	6.5
720.4	0.0045	0.021	7.5	7.5
720.6	0.018	0.025	4.5	3.5
720.9	0.0023	0.016	8.5	8.5
721.2	0.0011	0.012	9.5	9.5
721.7	0.015	0.027	5.5	4.5
722.7	0.025	0.035	7.5	6.5
722.9	0.011	0.026	6.5	5.5
723.3	0.013	0.014	2.5	1.5
724.0	0.0077	0.024	7.5	6.5
725.1	0.0046	0.021	8.5	7.5
726.0	0.016	0.032	8.5	7.5
726.2	0.0025	0.018	9.5	8.5
726.2	0.014	0.017	3.5	2.5
727.3	0.0012	0.014	10.5	9.5
728.4	5.4E-4	0.010	11.5	10.5
729.1	0.013	0.018	4.5	3.5
729.3	0.0092	0.028	9.5	8.5
731.9	0.010	0.018	5.5	4.5
732.6	0.0048	0.022	10.5	9.5
734.7	0.0071	0.016	6.5	5.5
735.9	0.0022	0.017	11.5	10.5

continued on next page

TABLE I – continued from previous page

Freq (cm^{-1})	Int. 38K (a.u.)	Int. 90K (a.u.)	J'	J"
737.5	0.0044	0.014	7.5	6.5
738.8	0.0015	0.012	9.5	10.5
739.1	9.6E-4	0.013	12.5	11.5
739.4	0.0031	0.015	8.5	9.5
740.0	0.0058	0.017	7.5	8.5
740.1	0.0024	0.011	8.5	7.5
740.7	0.0097	0.020	6.5	7.5
741.5	0.015	0.021	5.5	6.5
742.3	0.020	0.021	4.5	5.5
743.2	0.025	0.020	3.5	4.5
744.2	0.027	0.018	2.5	3.5
745.2	0.026	0.015	1.5	2.5
746.4	0.023	0.011	0.5	1.5
750.6	0.021	0.011	1.5	1.5
751.6	0.035	0.019	2.5	2.5
752.8	0.041	0.027	3.5	3.5
754.0	0.040	0.033	4.5	4.5
755.4	0.035	0.036	5.5	5.5
756.8	0.026	0.037	6.5	6.5
758.2	0.018	0.035	7.5	7.5
759.7	0.011	0.032	8.5	8.5
761.3	0.0058	0.027	9.5	9.5
762.9	0.0029	0.022	10.5	10.5
763.7	0.016	0.011	4.5	3.5
764.6	0.0013	0.017	11.5	11.5
766.3	5.2E-4	0.012	12.5	12.5

continued on next page

TABLE I – continued from previous page

Freq (cm^{-1})	Int. 38K (a.u.)	Int. 90K (a.u.)	J'	J"
767.1	0.017	0.014	5.5	4.5
770.7	0.015	0.016	6.5	5.5
774.3	0.012	0.017	7.5	6.5
777.9	0.0080	0.016	8.5	7.5
781.7	0.0049	0.015	9.5	8.5
785.4	0.0027	0.013	10.5	9.5
789.3	0.0013	0.010	11.5	10.5

TABLE II: Calculated frequencies and intensities based on fitted spectroscopic parameters of[4] of the Delta states

Freq (cm^{-1})	Int. 38K (a.u.)	Int. 90K (a.u.)	J'	J"
667.8	3.1E-4	0.010	11.5	11.5
669.1	6.8E-4	0.013	10.5	10.5
670.3	0.0013	0.015	9.5	9.5
671.5	0.0023	0.016	8.5	8.5
672.5	0.0035	0.016	7.5	7.5
673.5	0.0045	0.014	6.5	6.5
674.3	0.0050	0.012	5.5	5.5
685.7	9.1E-4	0.012	10.5	11.5
687.8	0.0022	0.017	9.5	10.5
688.2	5.9E-4	0.011	9.5	10.5
688.2	0.0051	0.012	6.5	5.5
689.6	0.0041	0.013	7.5	6.5
690.0	0.0046	0.022	8.5	9.5
690.5	0.0014	0.015	8.5	9.5

continued on next page

TABLE II – continued from previous page

Freq (cm^{-1})	Rel. Int 38K (a.u.)	Rel. Int 90K (a.u.)	J’	J”
690.8	0.0029	0.013	8.5	7.5
691.9	0.0018	0.013	9.5	8.5
692.1	0.0089	0.027	7.5	8.5
692.8	0.0028	0.020	7.5	8.5
692.9	1.0E-3	0.011	10.5	9.5
694.2	0.015	0.030	6.5	7.5
695.1	0.0052	0.024	6.5	7.5
696.4	0.022	0.032	5.5	6.5
697.3	0.0086	0.027	5.5	6.5
698.5	0.028	0.029	4.5	5.5
699.6	0.012	0.028	4.5	5.5
700.6	0.028	0.023	3.5	4.5
701.9	0.015	0.026	3.5	4.5
702.7	0.018	0.012	2.5	3.5
704.2	0.015	0.021	2.5	3.5
706.5	0.0095	0.011	1.5	2.5
710.2	0.14	0.081	2.5	2.5
710.2	0.19	0.12	3.5	3.5
710.2	0.18	0.14	4.5	4.5
710.3	0.14	0.14	5.5	5.5
710.3	0.093	0.13	6.5	6.5
710.3	0.056	0.11	7.5	7.5
710.3	0.030	0.092	8.5	8.5
710.4	0.015	0.070	9.5	9.5
710.4	0.0066	0.051	10.5	10.5
710.4	0.0027	0.036	11.5	11.5

continued on next page

TABLE II – continued from previous page

Freq (cm^{-1})	Rel. Int 38K (a.u.)	Rel. Int 90K (a.u.)	J'	J"
710.4	9.9E-4	0.024	12.5	12.5
710.4	3.4E-4	0.015	13.5	13.5
712.0	1.9E-4	0.012	12.5	12.5
712.0	5.5E-4	0.018	11.5	11.5
712.0	0.0014	0.027	10.5	10.5
712.0	0.0034	0.038	9.5	9.5
712.1	0.0073	0.051	8.5	8.5
712.1	0.014	0.066	7.5	7.5
712.1	0.025	0.079	6.5	6.5
712.1	0.039	0.089	5.5	5.5
712.1	0.053	0.094	4.5	4.5
712.2	0.064	0.090	3.5	3.5
712.2	0.064	0.075	2.5	2.5
712.2	0.047	0.049	1.5	1.5
715.5	0.42	0.21	2.5	1.5
715.6	0.067	0.065	1.5	0.5
717.7	0.36	0.20	3.5	2.5
717.9	0.070	0.073	2.5	1.5
719.9	0.29	0.19	4.5	3.5
720.1	0.065	0.077	3.5	2.5
722.0	0.21	0.17	5.5	4.5
722.4	0.055	0.076	4.5	3.5
724.2	0.14	0.15	6.5	5.5
724.6	0.041	0.071	5.5	4.5
726.4	0.086	0.12	7.5	6.5
726.9	0.027	0.063	6.5	5.5

continued on next page

TABLE II – continued from previous page

Freq (cm^{-1})	Rel. Int 38K (a.u.)	Rel. Int 90K (a.u.)	J'	J"
728.5	0.048	0.097	8.5	7.5
729.1	0.017	0.053	7.5	6.5
730.7	0.024	0.073	9.5	8.5
731.3	0.0091	0.042	8.5	7.5
732.9	0.011	0.054	10.5	9.5
733.6	0.0045	0.032	9.5	8.5
735.1	0.0049	0.038	11.5	10.5
735.8	0.0021	0.023	10.5	9.5
737.3	0.0019	0.025	12.5	11.5
738.0	8.4E-4	0.016	11.5	10.5
739.5	6.9E-4	0.016	13.5	12.5
740.2	3.2E-4	0.011	12.5	11.5
741.7	2.3E-4	0.010	14.5	13.5
747.3	0.017	0.014	4.5	4.5
748.1	0.020	0.021	5.5	5.5
748.9	0.018	0.026	6.5	6.5
749.9	0.014	0.029	7.5	7.5
750.9	0.0100	0.030	8.5	8.5
752.1	0.0060	0.028	9.5	9.5
753.3	0.0033	0.025	10.5	10.5
754.6	0.0016	0.021	11.5	11.5
756.0	6.8E-4	0.016	12.5	12.5
756.9	0.018	0.012	4.5	3.5
757.4	2.7E-4	0.012	13.5	13.5
759.8	0.021	0.017	5.5	4.5
762.8	0.020	0.021	6.5	5.5

continued on next page

TABLE II – continued from previous page

Freq (cm^{-1})	Rel. Int 38K (a.u.)	Rel. Int 90K (a.u.)	J'	J"
765.9	0.017	0.024	7.5	6.5
769.1	0.012	0.025	8.5	7.5
772.4	0.0079	0.024	9.5	8.5
775.8	0.0045	0.021	10.5	9.5
779.3	0.0023	0.018	11.5	10.5
782.9	0.0011	0.014	12.5	11.5
786.5	4.6E-4	0.011	13.5	12.5

REFERENCES

[1] R. N. Zare, *Angular Momentum* (Wiley, New York, 1988).

[2] M. C. V. Vroonhoven and G. C. Groenenboom, “Reassignment of the O_2 spectrum just below dissociation threshold based on *ab initio* calculations,” J. Chem. Phys. **117**, 5240–5251 (2002).

[3] D. Papoušek and M. Aliev, *Rotation and Vibrational-Rotational Spectra* (Elsevier, Amsterdam, 1982).

[4] S.-J. Tang, Y.-C. Chou, J. J.-M. Lin, and Y.-C. Hsu, “The bending vibrational levels of the acetylene cation: A case study of the Renner-Teller effect in a molecule with two degenerate bending vibrations,” J. Chem. Phys. **125**, 133201 (2006).
